# Raspberry bush – derived magnetic nanocomposites for water purification and microwave absorption

**DOI:** 10.1039/d6na00547k

**Published:** 2026-09-10

**Authors:** Bagher Aslibeiki, Mahsa Imani, Mahsa Mahmoodi, Sagnik Ghosh, Davide Peddis, Tapati Sarkar

**Affiliations:** a Faculty of Physics, University of Tabriz Tabriz Iran b.aslibeiki@tabrizu.ac.ir; b Department of Materials Science and Engineering, Uppsala University Box 35 Uppsala SE-75103 Sweden tapati.sarkar@angstrom.uu.se; c Department of Chemistry and Industrial Chemistry & Genova, INSTM RU, nM2-Lab, University of Genova 16146 Genova Italy; d Institute of Structure of Matter, National Research Council, nM2-Lab Via Salaria km 29.300, Monterotondo Scalo 00015 Roma Italy

## Abstract

Integrating heavy-metal remediation and electromagnetic wave (EMW) attenuation within a single, sustainable material system remains a critical challenge, primarily due to the difficulty of synergistically regulating adsorption-active porous architectures and charge-transport networks. Herein, we address this challenge by utilizing raspberry bush biomass, an abundant, renewable, and naturally microstructured precursor, to fabricate a hierarchical porous activated carbon framework decorated with magnetite nanoparticles (Fe_3_O_4_/AC). The inherent structural advantages of the raspberry-bush-derived carbon matrix provide abundant oxygen-containing surface functionalities and a tailored pore distribution, which serve the dual purpose of facilitating heavy-metal capture and modulating dielectric loss. For environmental remediation, the Fe_3_O_4_/AC nanocomposite achieved an exceptional Pb^2+^ removal efficiency of >99.5% with a maximum removal capacity of 249.24 mg g^−1^ under optimized conditions (pH 9, 30 min, and 50 mg L^−1^ initial concentration), demonstrating robust recyclability over four cycles. Thermochemical and kinetic modeling confirmed a spontaneous process governed by pseudo-second-order kinetics. For electromagnetic shielding, the nanocomposite exhibited a minimum reflection loss (RL_min_) of −30 dB at 16 GHz within the Ku band. This outstanding electromagnetic dissipation is governed by a magneto–dielectric synergistic effect, where the carbon framework drives interfacial polarization and conduction losses, while the Fe_3_O_4_ nanoparticles optimize impedance matching. This work highlights a sustainable, biomass-derived strategy for designing high-performance, multifunctional materials that can find applications in both water purification and electromagnetic wave absorption.

## Abstract 2

This study develops a sustainable biomass-derived Fe_3_O_4_/activated carbon nanocomposite that combines efficient Pb^2+^ removal with strong electromagnetic wave attenuation (RL_min_ of −30 dB at 16 GHz).

## Graphical abstract

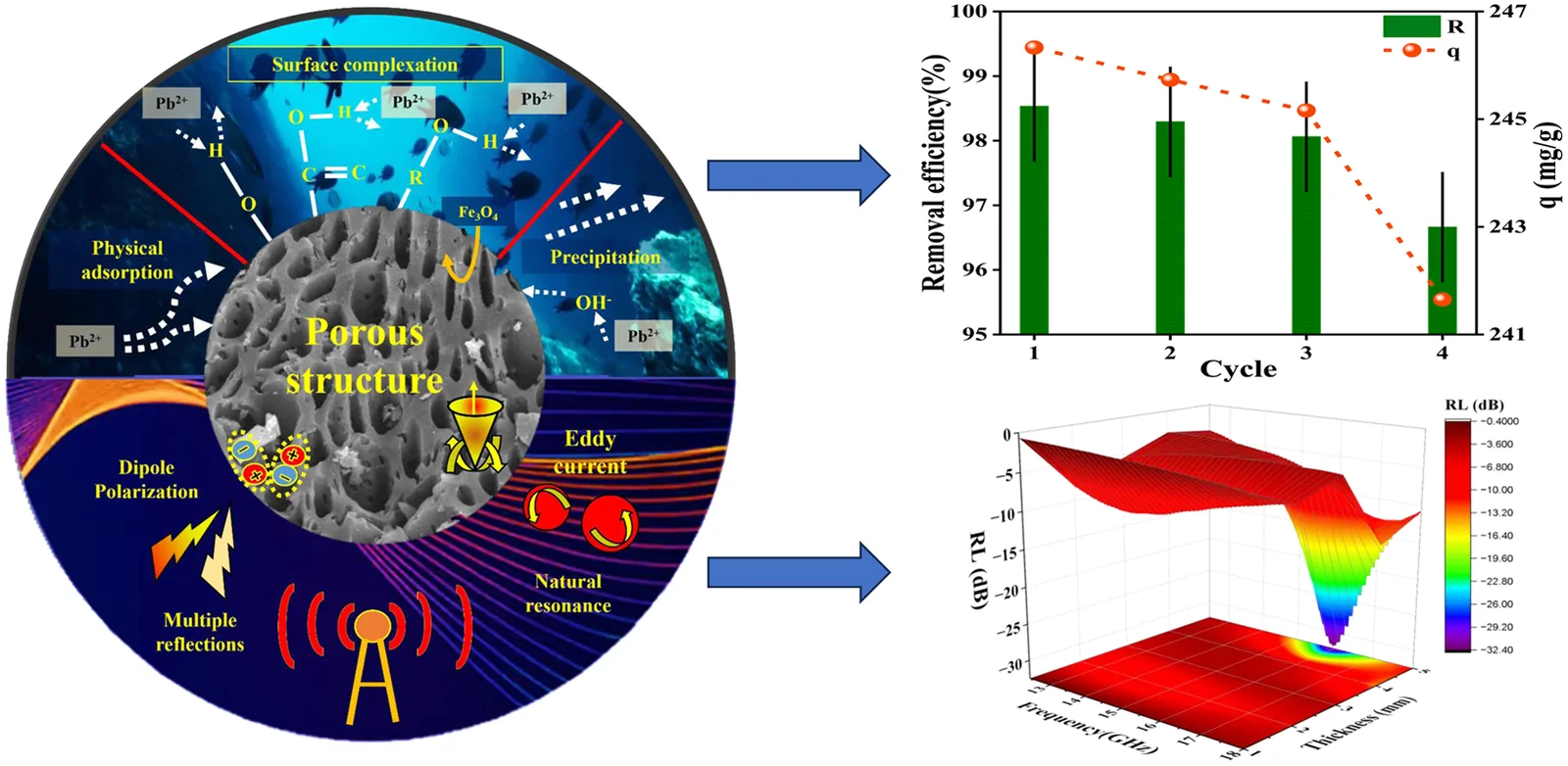

## 1 Introduction

Contaminated wastewater poses a significant threat to the environment and human health due to the presence of heavy metals such as lead, cadmium, chromium, and cobalt.^1^ These metals are non-biodegradable, persistent, and bioaccumulative, leading to severe ecological damage and adverse health effects. The rapid growth of industrial, agricultural, and domestic activities has considerably increased the release of heavy metals into aquatic environments, making efficient treatment technologies essential.^2^ Once introduced into the environment, heavy metals can migrate through soil and water, accumulate in living organisms, and enter the food chain, causing toxic, mutagenic, and carcinogenic effects even at low concentrations. Among various heavy metals, lead ions (Pb^2+^) are particularly hazardous and frequently detected in industrial wastewater, necessitating their effective removal. Adsorption is widely regarded as one of the most promising wastewater treatment methods due to its simplicity, cost-effectiveness, and environmental compatibility. In recent years, magnetic nanocomposites, especially using magnetite (Fe_3_O_4_), have gained considerable attention as adsorbents because of their high surface area, low toxicity, and easy separation from aqueous solutions by an external magnetic field.^3,4^ At the same time, activated carbon (AC) derived from biomaterials has emerged as a sustainable adsorbent owing to its porous structure, large surface area, and abundance of surface functional groups suitable for heavy metal adsorption.^5,6^

Beyond wastewater treatment, magnetic nanocomposites have also attracted increasing interest in electromagnetic wave (EMW) absorption applications.^7,8^ The growing use of wireless communication systems and radar technologies has intensified the demand for efficient microwave-absorbing materials, particularly in the Ku band (12.4–18 GHz), which is widely used in satellite communications and radar systems. Effective microwave absorbers should exhibit strong dielectric and magnetic losses, good impedance matching, broad absorption bandwidth, and lightweight characteristics. Recently, high-entropy ceramics (HECs) have emerged as a promising class of microwave-absorbing materials owing to their tailorable compositions, severe lattice distortion, and tunable dielectric and magnetic properties. Wang *et al.*^9^ demonstrated that magnetic Co incorporation into high-entropy carbide ceramics significantly enhances dielectric–magnetic loss synergy, resulting in excellent microwave attenuation performance. Similarly, Du *et al.*^10^ synthesized polymer-derived HEC–SiC biphasic ceramics, in which the introduction of nanosized SiC effectively improved impedance matching and interfacial polarization, leading to outstanding microwave absorption performance. These studies highlight the importance of optimizing composition, microstructure, and interfacial polarization to achieve efficient EMW attenuation.

In a nanocomposite containing magnetite nanoparticles and activated carbon, the Fe_3_O_4_ nanoparticles contribute to magnetic loss mechanisms such as natural resonance and eddy current loss, while activated carbon provides dielectric loss through interfacial polarization, dipolar polarization, and conductive loss.^11^ Therefore, the combination of Fe_3_O_4_ with activated carbon offers a synergistic effect, making Fe_3_O_4_/AC nanocomposites promising candidates for dual-functional applications, including both heavy metal removal and microwave absorption. In this study, a raspberry bush-derived magnetic nanocomposite is synthesized and investigated for its dual functionality. Compared with conventional Fe_3_O_4_/carbon composites, the novelty of this work lies not merely in combining a magnetic phase with a carbon matrix, but in the purposeful use of raspberry bush biomass as a naturally structured bioprecursor for simultaneously engineering porosity, surface chemistry, and electromagnetic response. Raspberry pruning residues have been recognized as a valuable lignocellulosic biomass resource. Recent studies have demonstrated their significant potential for conversion into functional materials such as biochar, activated carbon, and biosorbents, which are crucial for environmental remediation. Specifically, raspberry cane residues have emerged as promising precursors for both biochar-based pollutant removal and cost-effective heavy-metal adsorption, underscoring their environmental valorization within a circular economy framework.^12^ The BET results obtained in the current study (discussed later) further confirm that the derived nanocomposite possesses a highly accessible porous architecture, with a specific surface area of 68.19 m^2^ g^−1^, total pore volume of 0.4014 cm^3^ g^−1^, micropore volume of 0.3126 cm^3^ g^−1^, and an average pore diameter of approximately 2 nm. Such a pore network provides abundant adsorption sites for Pb^2+^ uptake while simultaneously prolonging the propagation path of electromagnetic waves, promoting multiple internal reflections and enhancing dielectric heterogeneity, thereby favoring electromagnetic energy dissipation. In this framework, raspberry bush is not simply a renewable carbon source, but a structure-directing biological precursor that enables the formation of a dual-functional porous network. Moreover, because raspberry bushes are seasonal and thorny, their above-ground biomass is commonly available as pruning residues at the end of the growth cycle, implying low ecological burden, no direct competition with arable land, and minimal need for dedicated cultivation. Meanwhile, Fe_3_O_4_ nanoparticles not only provide magnetic separability but also contribute magnetic loss and improved impedance matching, acting synergistically with the dielectric loss of the carbon framework. The combined effect is a multifunctional system capable of simultaneously capturing heavy-metal ions and attenuating microwave radiation in an efficient and sustainable manner.

The simultaneous targeting of heavy-metal contaminants and electromagnetic radiation in a single study provides a scientifically grounded response to two United Nations Sustainable Development Goals (SDGs). SDG 6 emphasizes clean water and sanitation for all, whereas the contamination of aquatic resources by heavy metals such as Pb, Cd, and Cr remains one of its major challenges. These pollutants not only harm aquatic ecosystems but also pose serious risks to human health through bioaccumulation and transfer along the food chain. In parallel, SDG 9 calls for resilient infrastructure, sustainable industrialization, and the adoption of clean and environmentally responsible technologies in industrial processes. In this context, the development of materials capable of treating industrial wastewater while also mitigating electromagnetic pollution from telecommunication and industrial equipment represents a practical contribution toward green and sustainable industry. Recent studies have confirmed this dual-function paradigm by demonstrating, for example, that a lignin/Fe_3_O_4_ composite can achieve a Pb(ii) adsorption capacity of 189 mg g^−1^ together with a reflection loss value (RL_min_) of −28.65 dB, indicating that adsorption and electromagnetic attenuation can be integrated within a single material platform.^13^ Likewise, an apple tree root-derived biochar/iron oxide triphasic nanocomposite reached a heavy-metal adsorption capacity of 731 mg g^−1^ and an RL_min_ of −61.7 dB with an effective absorption bandwidth of 7.8 GHz, further evidencing the strong synergy between surface chemistry and electromagnetic loss mechanisms.^14^ In addition, Ma *et al.* reported a ZIF-derived FeC-based nanostructure capable of simultaneously degrading antibiotics through an Electro-Fenton process and attenuating microwave radiation, confirming that engineered carbon/iron-containing systems can couple environmental remediation with electromagnetic-wave absorption.^15^ More recently, PAN/β-CD/MnO_2_ composite fibers were shown to exhibit highly efficient adsorption of heavy metals alongside effective electromagnetic-wave absorption, reinforcing the concept that multifunctional composites can address both aqueous pollution and electromagnetic interference within one unified design strategy.^16^ In parallel with these developments, recent advances in other fields of materials science have demonstrated that the integration of complementary functionalities within a single material system is a widely applicable and powerful strategy. For instance, in the field of microwave absorption, the narrow effective absorption bandwidth (EAB) of dielectric loss-type materials has been optimized through innovative fabrication approaches. One study reported a TiO_1.81_/Al_2_O_3_ coating fabricated by supersonic atmospheric plasma spraying (SAPS) of Ti_2_AlC/Al_2_O_3_ powders followed by oxidation at 950 °C, which exhibited almost twice the widest EAB reported among all dielectric loss-type materials. The *in situ* double decomposition mechanism transformed Ti_2_AlC into O-vacancy-defected TiO_1.81_ (30 nm) and Al_2_O_3_ (200–500 nm), significantly enhancing interfacial polarization due to the formation of abundant nano-heterointerfaces.^17^ Other emerging fields have also benefited from multifunctional integration, for example, in permanent magnets,^18^ ferroelectric materials,^19^ polymer dielectrics,^20^ antiferromagnetic spintronics,^21^ and magnetic sensor calibration.^22^ These diverse examples collectively highlight a broader paradigm: the rational design of composition, microstructure, and interfacial architecture is a powerful and generalizable strategy for creating multifunctional materials with outstanding performance. This paradigm aligns closely with the motivation of the present work, in which a biomass-derived magnetic nanocomposite is intentionally engineered to simultaneously address heavy-metal water pollution and EMW interference. Taken together, these findings confirm that the intrinsic synergy between magnetic and dielectric phases yields performance that transcends the arithmetic sum of individual functionalities, reinforcing the viability of dual-purpose materials for sustainable environmental applications.

## 2 Materials and methods

### 2.1. Chemicals

Metal salts, including ferrous chloride tetrahydrate (FeCl_2_·4H_2_O) and ferric chloride hexahydrate (FeCl_3_·6H_2_O) (Merck), were used to prepare ferrite nanoparticles. Sodium hydroxide (NaOH, Merck) was employed as the precipitating agent. Zinc chloride (ZnCl_2_, Merck) was used as the chemical activating agent for the carbon derived from raspberry bush biomass. Hydrochloric acid (HCl, Merck) and deionized water were used for washing and as solvents, respectively. Sodium nitrate (NaNO_3_, Merck) was used to determine the point of zero charge (pH_PZC_). Lead nitrate (Pb(NO_3_)_2_, Merck) was used to prepare Pb^2+^ stock solutions for batch adsorption experiments.

### 2.2. Preparation of raspberry bush-derived activated carbon (AC)

Initially, the raspberry bushes were cut into small pieces, thoroughly washed with deionized water to remove impurities, and air-dried at room temperature for several days. The dried biomass was then chemically impregnated with a 2 M zinc chloride (ZnCl_2_) solution at a mass ratio of 1 : 1 (biomass : ZnCl_2_) and stirred for 24 h. After impregnation, the material was dried using a heater to remove excess moisture.

The dried sample was subsequently placed in a furnace at 110 °C for 5 h to ensure complete dehydration. Pyrolysis was then carried out in a tubular furnace at 600 °C for 2 h under nitrogen flow. After cooling to room temperature, the resulting carbon material was treated with 0.1 M hydrochloric acid (HCl) for 24 h to remove residual activating agents and inorganic impurities. The sample was then filtered and washed repeatedly with deionized water until a neutral pH was achieved. Finally, the activated carbon was dried completely at room temperature for several days and stored for further use (Fig. 1).

**Fig. 1:**
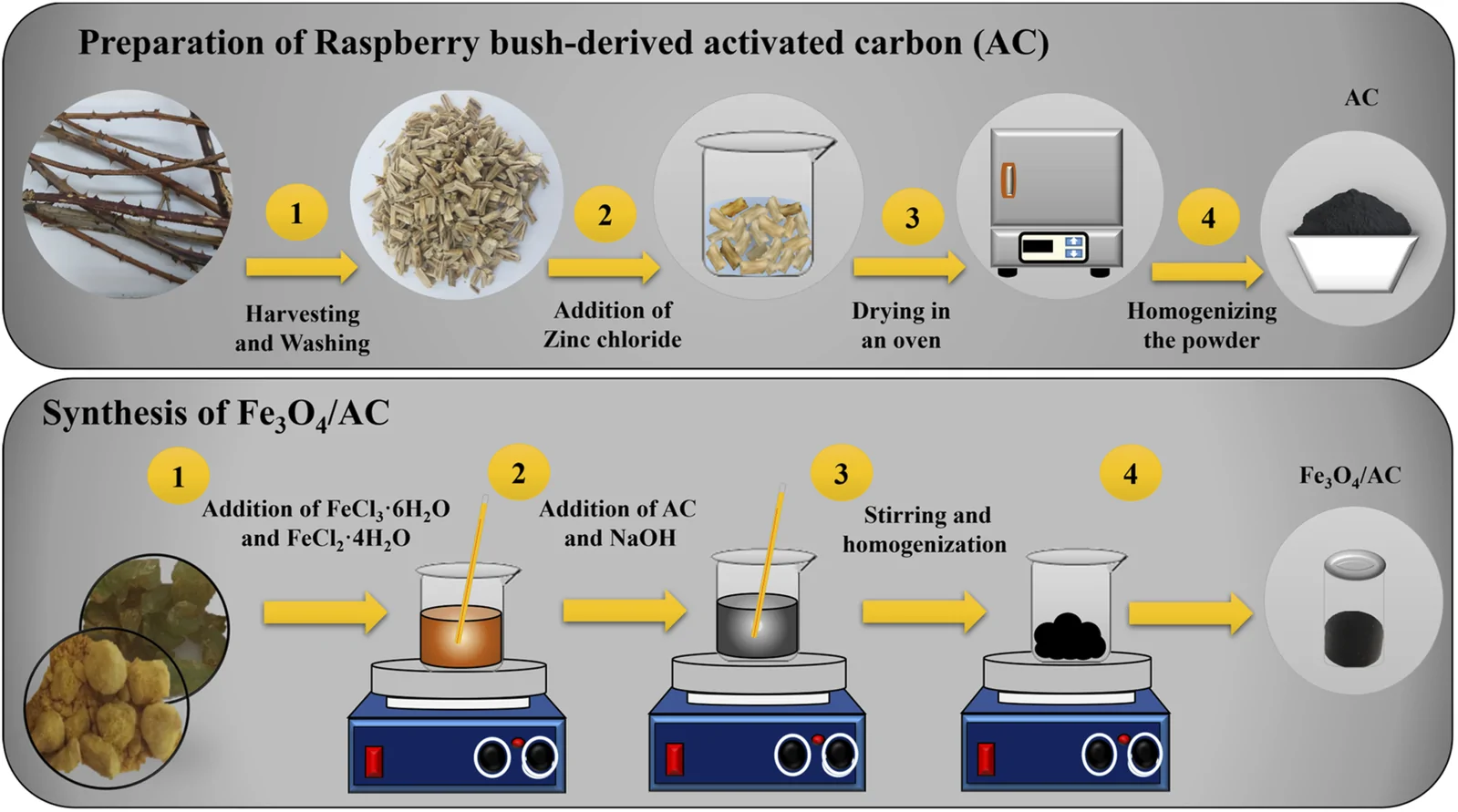
Schematic representation of the preparation process of AC and Fe_3_O_4_/AC.

### 2.3. Preparation of Fe_3_O_4_/AC nanocomposite

The activated carbon was first sieved through 210 and 297 µm meshes to obtain a uniform and homogeneous powder. Ferrous chloride tetrahydrate (FeCl_2_·4H_2_O) and ferric chloride hexahydrate (FeCl_3_·6H_2_O) were dissolved in deionized water, and the solution temperature was raised to 80 °C. The sieved activated carbon was then added to the solution and stirred for 15 min to ensure uniform dispersion. Subsequently, an aqueous sodium hydroxide (NaOH) solution was gradually added to increase the pH. After 30 min of reaction, the Fe_3_O_4_/AC nanocomposite precipitated. The resulting product was collected and washed three times with deionized water to remove residual impurities.

The Fe_3_O_4_ : AC mass ratio was fixed at 25 : 75 based on the results of our previous experimental studies. The relatively high AC content was intentionally selected because the carbonaceous phase plays a dominant role in heavy-metal removal and dielectric microwave attenuation, whereas Fe_3_O_4_ was incorporated primarily to provide magnetic separability and contribute to the magnetic response of the composite. Our previous results also showed that increasing the ferrite fraction at the expense of the carbon content did not necessarily improve microwave absorption. In particular, the ferrite phase alone exhibited a relatively limited microwave absorption performance of approximately −13 dB. Therefore, the 25 : 75 (Fe_3_O_4_ : AC) ratio was selected by considering the heavy-metal adsorption performance, dielectric loss, magnetic separability, and material cost.^6,23,24^

### 2.4. Characterization techniques

A scanning electron microscope (SEM, Zeiss LEO 1550) equipped with energy-dispersive X-ray spectroscopy (EDS) was used to examine the surface morphology and elemental composition of the samples. X-ray diffraction (XRD) patterns were obtained using a Siemens D5000 diffractometer to identify the crystalline phases. Raman spectra were recorded using a Horiba Jobin-Yvon LabRAM HR800 spectrometer. The magnetic properties of the samples were measured using a vibrating sample magnetometer (VSM, Lake Shore model 4701) with a maximum applied magnetic field of 18 kOe. X-ray photoelectron spectroscopy (XPS, PHI QUANTERA II) employing Al Kα radiation was used to analyze the surface elemental composition and chemical states. Brunauer–Emmett–Teller (BET) surface area and porosity analyses were conducted using a BELSORP MINI II (Japan).

The concentration of metal ions in aqueous solutions was determined using an inductively coupled plasma optical emission spectrometer (ICP 5000 DV). For electromagnetic characterization, the Fe_3_O_4_/AC nanocomposite was blended with paraffin wax at a weight ratio of 70 : 30 (paraffin : nanocomposite). The mixture was then molded into a specimen with a thickness of 1 mm, matching the dimensions required for the WR-62 Ku-band waveguide holder. Microwave measurements in the Ku band were carried out using a Keysight N5227A PNA vector network analyzer, calibrated in transmission-line mode with a Keysight N9911X-WR62 mechanical calibration kit. The complex permittivity and permeability were determined using the Nicolson–Ross–Weir (NRW) method through the Keysight N1500A Materials Measurement Suite (2020). The reflection loss (RL) values for absorber thicknesses of 1–5 mm were subsequently calculated in MATLAB from the measured electromagnetic parameters using transmission-line theory. The Radar Cross-Section (RCS) simulation was performed using Computer Simulation Technology (CST) Studio Suite.

### 2.5. Pb(ii) removal experiments

The batch experiments were conducted using aqueous solutions prepared from lead nitrate (Pb(NO_3_)_2_, Merck). Stock solutions were prepared by dissolving Pb(NO_3_)_2_ in deionized water, and the solution pH was adjusted using NaOH and HCl solutions. Then 15 mL of the lead nitrate solution was transferred into a separate beaker, and a predetermined amount of the composite was added. The mixture was stirred mechanically for a specified contact time to ensure adequate interaction between the composite and the Pb^2+^ ions.

In the next step, the solid and liquid phases were separated by a magnet, and aliquots of the supernatant were collected for analysis. The remaining Pb^2+^ concentration in the solution was determined using inductively coupled plasma optical emission spectrometry (ICP-OES). The adsorption capacity and removal efficiency were calculated using eqn (1) and (2), respectively.1
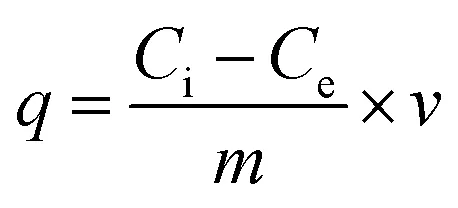
2
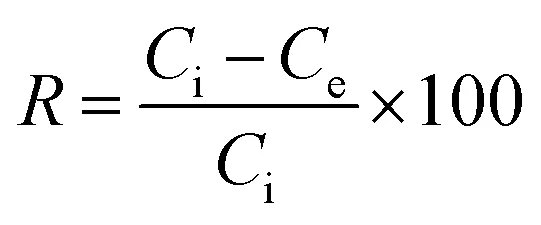
where, *C*_i_ and *C*_e_ are the initial and equilibrium concentrations of lead (mg L^−1^), respectively. *m* is the mass of the adsorbent (g), and *v* is the volume of the solution (L). To obtain the optimal pH, experiments were conducted at different pH values (3, 5, 7, 9, 11). For this, 15 cm^3^ of lead nitrate solution with an initial concentration of 50 mg L^−1^ was separated and adjusted to the desired pH by adding sodium hydroxide and hydrochloric acid to achieve basic and acidic pH values, respectively. A pH meter was used to monitor the pH values. Next, a predetermined amount of adsorbent was added to this solution and placed under a mechanical stirrer at room temperature. Finally, the adsorbent was separated from the solution, and the adsorption capacity and removal efficiency of the lead solution at different pH values were determined through ICP analysis. To investigate the effect of contact time, different times (3, 10, 20, 30, 50, 70 min) were tested. First, 15 cm^3^ of lead(ii) nitrate solution was brought to the optimal pH; next, the adsorbent was added to the solution and stirred at room temperature. Then, the adsorption capacity and removal efficiency were determined using ICP analysis to establish the optimal contact time. To achieve the optimal adsorbent dose, different amounts (1, 2, 3, 4, 5, 6 mg) of the composite were added to 15 cm^3^ lead nitrate solution at the optimal pH and stirred for the optimal contact time. Then the adsorbent was separated from the solution, and the optimal adsorbent dose was determined through ICP analysis. To determine the optimal initial concentration for lead removal, different concentrations (50, 110, 160, 210 mg L^−1^) were tested. Lead nitrate solution was prepared with the intended concentrations, and the adsorbent was added to the solution at the optimal pH at room temperature and placed under mechanical stirring. The liquid and solid phases were then separated, and the optimal initial concentration of the solution was determined using ICP analysis. To examine the impact of temperature on lead removal efficiency, experiments were conducted at temperatures of 306, 316, 326, and 336 K. The adsorbent was added to 15 mL of lead nitrate solution with an initial concentration of 50 mg L^−1^ at different temperatures and placed under mechanical stirring. The optimum temperature of the solution was determined through ICP analysis. To evaluate the reusability of the nanocomposite, the adsorbent was added to a Pb(ii) nitrate solution under the previously optimized conditions (initial concentration and pH) at room temperature with continuous mechanical stirring. After the first adsorption cycle, the adsorbent was separated from the solution. A portion of the recovered material was collected for further characterization analysis, while the remaining adsorbent was reused in the subsequent adsorption runs. This procedure was repeated for 4 consecutive cycles under the same experimental conditions. It should be noted that the experiments in this study were conducted in a single-ion Pb^2+^ system, which was a deliberate choice to isolate the intrinsic kinetic, isothermal, and thermodynamic behavior of the Fe_3_O_4_/AC nanocomposite without the interfering effects of ion competition. Real wastewater streams, however, typically contain coexisting cations such as Cd^2+^, Cu^2+^, Zn^2+^, and Ni^2+^. Based on established adsorption chemistry, Pb^2+^ is generally expected to retain a competitive advantage over these ions on oxygen-donor-rich surfaces such as activated carbon. Among common divalent heavy-metal ions, Pb^2+^ possesses one of the largest ionic radii (∼1.19 Å) and the lowest hydration enthalpy, which lowers the energetic penalty for dehydration during surface complexation and favors stronger, more covalent-character bonding with hydroxyl and carboxyl surface groups relative to smaller, more strongly hydrated ions such as Ni^2+^ and Zn^2+^. This behavior is consistent with the higher position of Pb^2+^ in reported selectivity series for oxygen-functionalized carbon and biochar-based adsorbents.

### 2.6. Adsorption kinetics models

Adsorption kinetics involves studying the speed of the adsorption process. Pseudo-first-order (PFO), pseudo-second-order (PSO) kinetics, and intraparticle diffusion models are commonly used to study the rate and mechanism of the adsorption process. The PFO linear equation is as follows:3
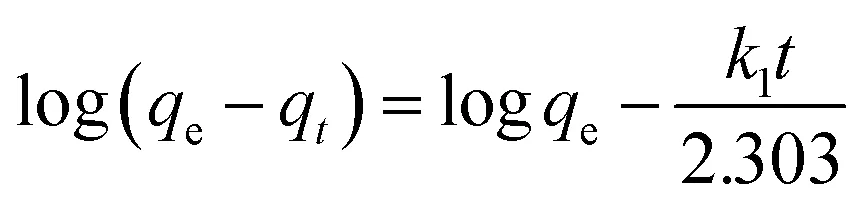


The PSO equation is as follows:4
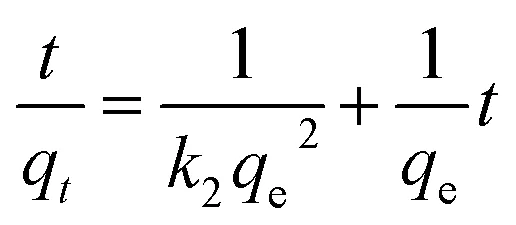
where, *q*_e_ (mg g^−1^) is the equilibrium adsorption capacity and *k*_1_ (min^−1^) and *k*_2_ (g mg^−1^ min^−1^) are the rate constants of the PFO and PSO models, respectively.

The intraparticle diffusion equation is as follows:

5 *q*_*t*_ = *k*_d_*t*^1/2^ + *C*

where, *k*_d_ (mg g^−1^ min^−1/2^) is the intraparticle diffusion rate constant, and *C* (mg g^−1^) is related to the boundary layer thickness.

### 2.7. Adsorption isotherms

Adsorption isotherms describe how an adsorbate distributes itself between the liquid phase and a solid surface at a constant temperature. Several established models can be applied to characterize these equilibrium dynamics. The linear form of the Freundlich isotherm is as follows:^25^6
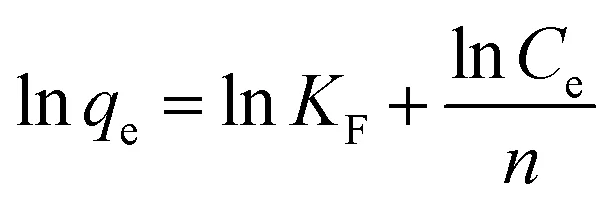


Freundlich's constant (*K*_F_) is related to the adsorption capacity of the adsorbent. The empirical parameter 1/*n* is related to the adsorption power, which is different from the heterogeneity of the material. When the value of 1/*n* is between 0.1 and 1.0, the adsorption process is considered favorable.

The linear form of the Langmuir isotherm can be expressed based on the following equation:7
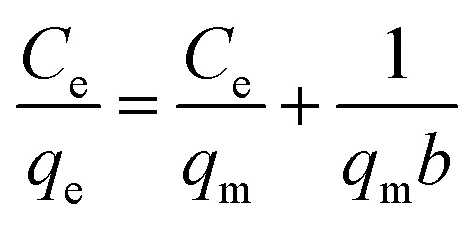
where, *q*_m_ is the maximum biosorption capacity (mg g^−1^), and *b* is the Langmuir constant (L mg^−1^). The Temkin isotherm model is as follows:

8 *q*_e_ = *B* ln *K*_T_ + *B* ln *C*_e_

where, *B* = *RT*/*b*, in which *R* is the universal gas constant (8.314 J mol^−1^ K^−1^), *T* is the absolute temperature, *b* (J mol^−1^) is the compliance constant obtained from the heat of adsorption, and *K*_T_ (L mg^−1^) is the equilibrium constant.

### 2.8. Nonlinear isotherm models

The nonlinear Langmuir isotherm model is extensively employed to interpret adsorption equilibrium data, assuming monolayer coverage over a structurally homogeneous surface with a finite number of identical binding sites.9
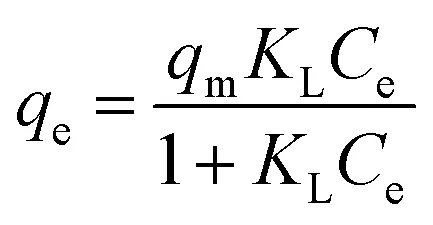
where, *q*_m_ (mg g^−1^) is the theoretical maximum adsorption capacity, and *K*_L_ (L mg^−1^) is the Langmuir affinity constant representing the interaction between adsorbate molecules and the active sites of the adsorbent.

The Freundlich isotherm is recognized as one of the most commonly applied models for describing adsorption behavior. The following equation expresses the nonlinear form of this model.10
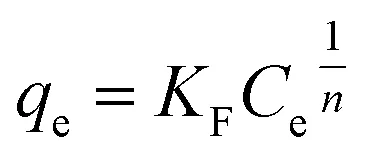
where, *K*_F_ and *n* represent the Freundlich constants.

The Temkin isotherm model is used to describe adsorbate–adsorbent interactions and the variation in adsorption heat during the adsorption process.11
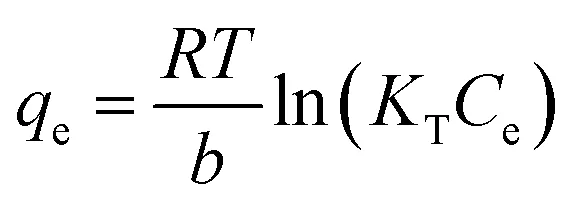
where, *K*_T_ (L g^−1^) and *B* (J mol^−1^) are the characteristic Temkin constants.

To complement the linear analysis and minimize potential parameter distortions caused by linear transformations, the experimental kinetic data were also analyzed using the non-linear forms of the PFO and PSO models. The non-linear PFO rate equation is expressed as:

12 *q*_*t*_ = *q*_e_ (1 − e^−*k*_1_*t*^)

where, *q*_e_ and *q*_*t*_ (mg g^−1^) represent the adsorption capacities at equilibrium and at time *t* (min), respectively, and *k*_1_ (min^−1^) is the PFO rate constant.

The non-linear PSO kinetic model is formulated as:13
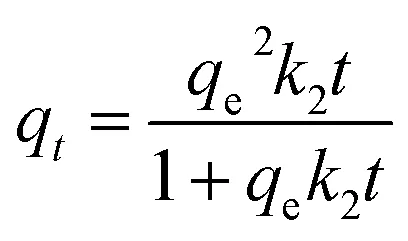


The parameters for both linear and non-linear regression models were calculated. To evaluate the goodness-of-fit and select the most appropriate model, the coefficient of determination (*R*^2^), residual analysis, and the corrected Akaike Information Criterion (AICc) were utilized. The AICc was calculated using the following equation:14
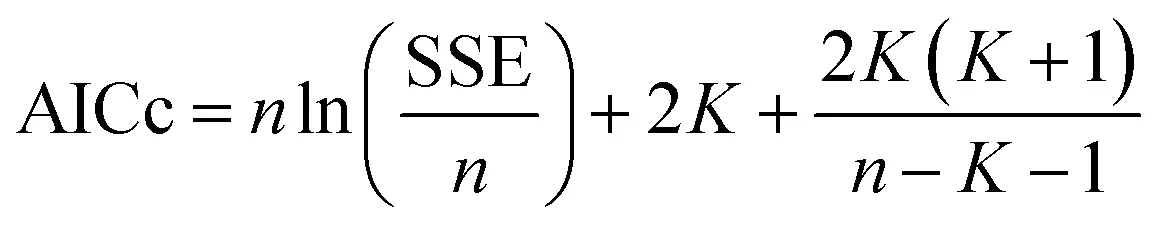
where, *n* is the number of experimental data points (*n* = 6), SSE is the sum of squared errors, and *K* represents the number of model parameters plus one.

### 2.9. Thermodynamics study

Thermodynamic analysis of the adsorption process was performed to evaluate the feasibility and spontaneity of Pb(ii) removal using the ferrite-activated carbon nanocomposite, and to gain insight into the interactions between lead ions and the adsorbent surface. The emission coefficient (*K*_L_) in each function is obtained using the following equation:15
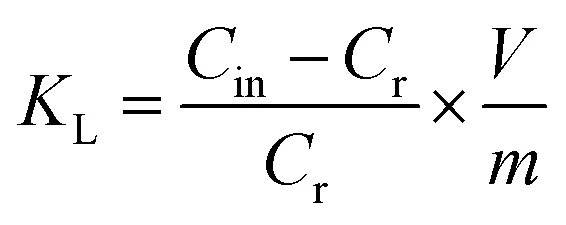
where, *C*_in_ and *C*_r_ represent the initial and equilibrium concentrations of Pb(ii) in solution (mg L^−1^), *V* is the solution volume (L), *m* is the mass of adsorbent (g), and *K*_L_ is expressed in units of L g^−1^. This coefficient provides insight into the distribution of lead ions between the solution and the adsorbent, and its value is used as a basis for further thermodynamic analysis.

Van't Hoff equation was used to establish the temperature-dependent correlation of *K*_L_ with changes in enthalpy (Δ*H*°) and entropy (Δ*S*°), which is as follows:16
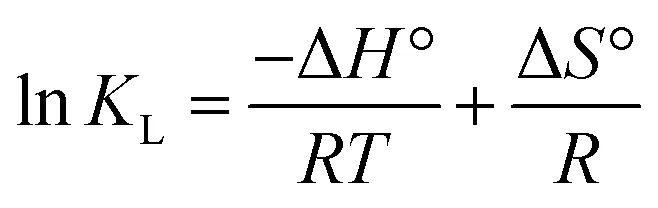


Lastly, Gibbs free energy (Δ*G*°) was obtained using the following equation:

17 Δ*G* = Δ*H*° − *T*Δ*S*°

## 3 Results and discussion

As shown in Fig. 2a, the activated carbon exhibits a porous structure with well-developed mesoporous and microporous features. In contrast, the FESEM image of the Fe_3_O_4_/AC nanocomposite in Fig. 2b reveals a rough surface morphology with larger, bulky agglomerates, which can be attributed to the adhesion and aggregation of Fe_3_O_4_ nanoparticles on the carbon surface.^26,27^ The observed surface modifications of the Fe_3_O_4_/AC nanocomposite can be attributed to the incorporation of magnetic Fe_3_O_4_ nanoparticles within the porous structure of the activated carbon, as confirmed by elemental mapping. The N_2_ adsorption–desorption isotherms and the corresponding BET plot, shown in Fig. 2c and d, reflect the effective surface area of the material. The analysis indicates an average pore diameter of approximately 2 nm, consistent with the sample's mesoporous nature. From the BET experiment and *t*-plot method (Fig. 2e), the micropore volume (*V*_1_) and micropore surface area (*A*_1_) were determined to be 0.3126 cm^3^ g^−1^ and 37.97 m^2^ g^−1^, respectively. The pore size distribution (1–100 nm) in Fig. 2f shows that the incremental pore volume decreases with increasing pore diameter. This analysis enables accurate determination of pore diameter, pore volume, and pore size distribution. The total pore volume of the sample was found to be 0.4014 cm^3^ g^−1^, with a corresponding specific surface area of 68.19 m^2^ g^−1^.

**Fig. 2:**
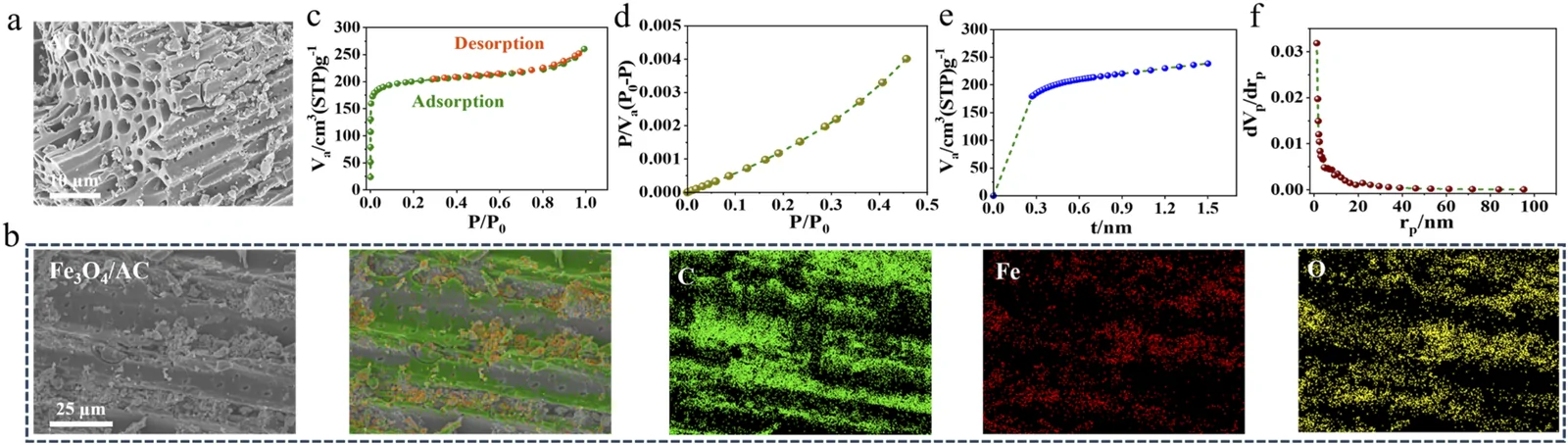
FESEM image of (a) raspberry bush-derived activated carbon (AC), (b) Fe_3_O_4_/AC composite, and corresponding elemental mapping, (c) nitrogen adsorption–desorption isotherms, (d) BET surface area, (e) *t*-plot, and (f) pore size distribution analysis for Fe_3_O_4_/AC nanocomposite.

### 3.1. Adsorption kinetics

The pH drift method was employed to determine the point of zero charge (pH_PZC_) of the Fe_3_O_4_/AC nanocomposite. In this procedure, 30 mL of deionized water containing 0.1 M sodium nitrate was prepared as the background electrolyte. The initial pH of the solution was adjusted over the range of 1–13 at intervals of 2 using dilute HCl or NaOH solutions. Subsequently, 0.012 g of the Fe_3_O_4_/AC nanocomposite was added to each solution, and the suspensions were stirred for 10 h until the pH reached equilibrium. The final pH values were then recorded. Determination of the pH_PZC_ provides valuable information regarding the suitable pH range for the adsorption of anionic and cationic species on the nanocomposite surface.^3^ For pH_PZC_ determination, the final pH values were plotted *versus* initial pH values. The intersection point of the curves with the line pH_final_ = pH_initial_ was identified as the pH_PZC_, which was found to be 3.77 for activated carbon and 7.03 for the Fe_3_O_4_/AC nanocomposite (Fig. 3a). When the pH is lower than pH_PZC_ (pH < pH_PZC_), surface functional groups become protonated, resulting in a positively charged surface. This positive surface charge leads to electrostatic repulsion between the adsorbent and positively charged heavy metal ions, thereby reducing adsorption efficiency. Consequently, the variations in adsorption efficiency observed in Fig. 3a could be attributed to non-electrostatic interactions. The high adsorption efficiency is likely related to the presence of surface hydroxyl groups, which provide active sites for binding Pb^2+^ ions. Conversely, when the solution pH exceeds pH_PZC_ (pH > pH_PZC_), the surface acquires a net negative charge, which enhances electrostatic attraction between the adsorbent surface and heavy metal ions, leading to increased adsorption efficiency.^14,28^

**Fig. 3:**
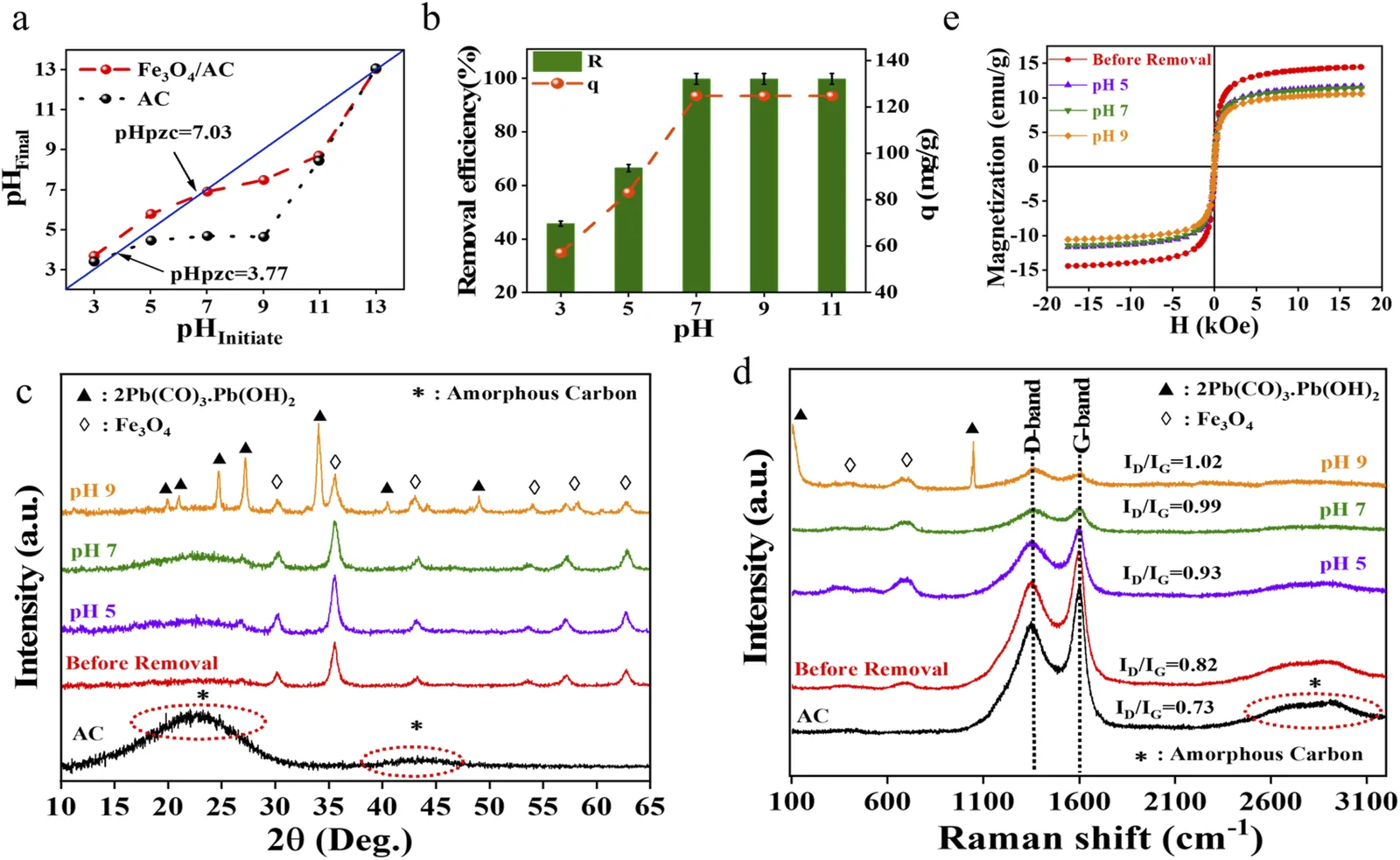
(a) pH_PZC_ of AC and Fe_3_O_4_/AC nanocomposite, (b) effect of pH on removal efficiency, (c) XRD patterns, (d) Raman spectra, and (e) magnetic hysteresis loops of the adsorbent, before and after Pb^2+^ removal at pH of 5, 7, and 9.

#### 3.1.1. Effect of pH

The pH is one of the most critical parameters influencing the removal process, as it affects the degree of ionization, the surface charge of the adsorbent, and the removal efficiency of heavy metal ions. The effect of pH on Pb^2+^ removal from contaminated water was investigated by maintaining a constant initial metal ion concentration of 50 mg L^−1^, a contact time of 30 minutes, and an adsorbent dosage of 6 mg. The solution pH was adjusted using dilute HCl and NaOH solutions. As shown in Fig. 3b, the removal of Pb^2+^ ions was examined over a pH range of 3–11. Under acidic conditions, metal ions remain more stable and exhibit reduced adsorption due to competition with protons for active adsorption sites. In contrast, increasing the solution pH leads to decreased metal ion stability and enhanced adsorption efficiency, thereby facilitating more effective removal of Pb^2+^ ions from the aqueous phase.^29^ As the solution pH increases, insoluble lead compounds, such as lead carbonate, may form in the aqueous phase. The formation of these precipitates reduces the availability of free Pb^2+^ ions for interaction with the nanocomposite surface, resulting in a decrease in the apparent removal efficiency of lead ions.^30,31^ The maximum removal efficiency of >99.5% was observed at a solution pH of 9, corresponding to a removal capacity of 124.74 mg g^−1^. This observation is consistent with the pH_PZC_ results, which indicate that at higher pH values, the surface of the adsorbent carries a net negative charge. The negative surface charge reduces electrostatic repulsion with Pb^2+^ ions, enhancing the attraction between the adsorbent and the metal ions, and thereby increasing both the removal efficiency and the adsorption capacity.^32^ To validate the results obtained from the batch experiments, complementary characterizations were performed under acidic, neutral, and alkaline conditions.

The XRD patterns of the AC and the nanocomposite powder before and after batch experiments at three selected pH values (5, 7, and 9) are shown in Fig. 3c. While the AC shows an amorphous phase structure demonstrating two broad peaks around 15°–30° and 40°–50°, the characteristic crystalline peaks of Fe_3_O_4_ were identified at 2*θ* values of 30.26°, 35.72°, 43.27°, 53.87°, 57.50°, and 63.06°, corresponding to the (220), (311), (400), (422), (511), and (440) planes of the cubic spinel structure with space group *Fd*3̄*m* (ICDD 0315-088-01).

Given that the ferrite nanoparticles exhibit a spinel cubic structure, the lattice constant (*a*) and the unit cell volume (*V*_uc_) were calculated using the following relations:

18 *a* = *d* (*h*^2^ + *k*^2^ + *l*^2^)^1/2^

19 *V*_uc_ = *a*^3^

where, *d* is the distance between the Bragg planes and (*hkl*) are the Miller indices of the lattice plane. The average crystallite size (*D*) was calculated using the Debye–Scherrer equation:20
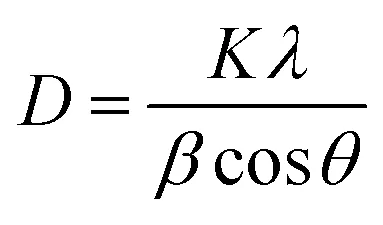
where, *K* is the shape factor (0.90), *λ* is the X-ray radiation wavelength (0.15406 nm), *β* is the full width at half maximum (FWHM) intensity, and *θ* is the Bragg diffraction angle.^33^ The lattice constant (*a*) was determined to be 8.37 Å, with a corresponding unit cell volume (*V*_uc_) of 587.43 Å^3^, and the crystallite size *D* was found to be 19.18 nm. While the XRD results of the adsorbent at pH 5 and 7 show similar results as that for before adsorption, at pH 9, additional diffraction peaks at (110), (104), and (015) were observed, corresponding to the formation of white lead (2PbCO_3_·Pb(OH)_2_) precipitate, which indicates the presence of lead carbonate and lead hydroxide species on the adsorbent surface. At neutral pH 7, although no crystalline Pb phases were detected, the removal efficiency remains high (see Fig. 3b), likely due to surface adsorption and ion-exchange mechanisms that do not produce detectable crystalline phases.

Fig. 3d shows the Raman spectra of the AC and adsorbent before and after Pb^2+^ removal at different pH values. The activated carbon exhibits two prominent peaks at approximately 1349 and 1602 cm^−1^, corresponding to the D and G bands, respectively.^34^ The intensity ratio of the D band (*I*_D_) to the G band (*I*_G_) is commonly used to assess the degree of structural disorder in carbon-based materials. Additional peaks observed at 391 and 688 cm^−1^ in the composite sample are attributed to the Fe_3_O_4_ phase. In addition, the observed peaks at around 100 and 1048 cm^−1^ for the adsorbent at pH 9 indicate the formation of 2PbCO_3_·Pb(OH)_2_,^35^ consistent with the XRD results. At pH ≥ 9, the formation of basic lead carbonate (2PbCO_3_·Pb(OH)_2_) becomes thermodynamically favorable, as confirmed by the XRD and Raman analyses of the spent adsorbent. In the presence of the Fe_3_O_4_/AC nanocomposite, however, the abundant oxygen-containing functional groups (–OH and –COOH) act as preferential nucleation and coordination sites for Pb(ii), promoting surface complexation followed by localized heterogeneous precipitation. Therefore, Pb(ii) removal at pH 9 results from the combined contribution of chemical complexation and surface-induced precipitation rather than homogeneous precipitation in the bulk solution.

Since the pH affects the adsorption efficiency, it can also influence the magnetic properties of the composite. Fig. 3e shows the magnetic hysteresis curves of the adsorbent before and after the batch experiments at pH 5, 7, and 9. The saturation magnetization (*M*_S_) of the Fe_3_O_4_/AC nanocomposite before Pb^2+^ removal is 14.44 emu g^−1^. After removal, the *M*_S_ decreased to 11.62, 11.44, and 10.58 emu g^−1^ at pH 5, 7, and 9, respectively. The reduction in saturation magnetization at pH 5 and 7 is attributed to the adsorption of lead ions on the nanocomposite surface. At pH 9, the formation of lead carbonate precipitate is likely responsible for the additional decrease in *M*_S_.^36^

### 3.2. Contact time and kinetic studies

To determine the optimal contact time for Pb^2+^ removal, batch experiments were conducted at an initial Pb^2+^ concentration of 50 mg L^−1^, an adsorbent dose of 6 mg, and a pH of 9, with measurements taken at different time intervals. As shown in Fig. 4a, the lead removal percentage increased with increasing contact time, reflecting the availability of vacant adsorption sites on the nanocomposite surface. Equilibrium was reached after approximately 30 minutes, with a maximum removal efficiency of >99.5% and an adsorption capacity of 124.75 mg g^−1^. A slight decrease in removal efficiency was observed after this time, which could be attributed to desorption or redistribution of Pb^2+^ ions from the adsorbent surface.

**Fig. 4:**
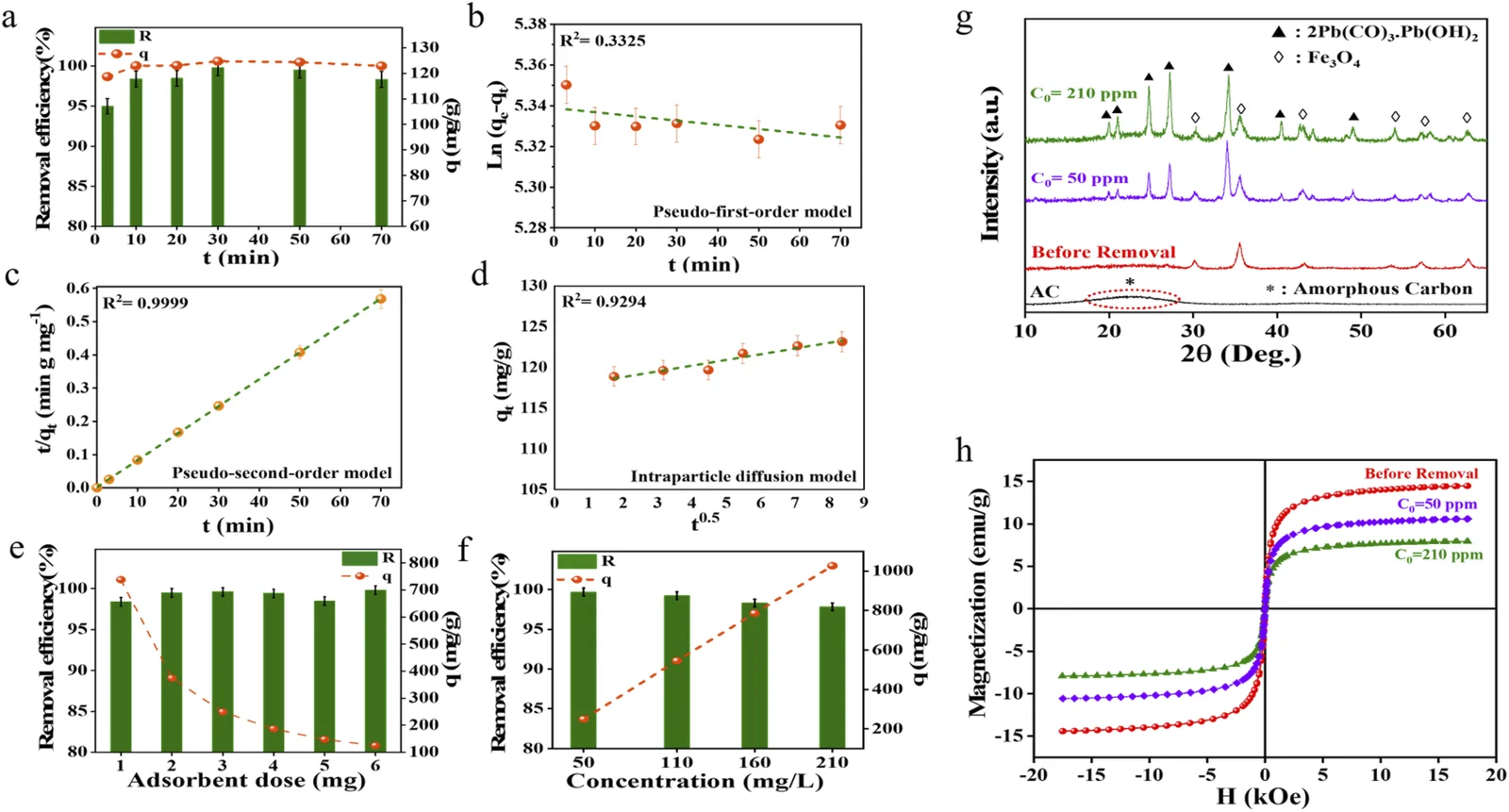
(a) Effect of contact time on *R* and *q* values. Fitting results of experimental data with (b) PFO, (c) PSO, and (d) intraparticle diffusion models, (e) effect of adsorbent dosage, (f) initial ion concentration on removal efficiency and capacity, (g) XRD patterns, and (h) magnetic hysteresis loops of the Fe_3_O_4_/AC composite before and after adsorption at concentrations of 50 and 210 ppm.

The adsorption kinetics of Pb^2+^ onto Fe_3_O_4_/AC were further analyzed using PFO and PSO kinetic models. The suitability of each model for describing the adsorption process was evaluated based on the coefficient of determination (*R*^2^).^37^

The PFO model, also known as the Lagergren rate equation, describes adsorption kinetics assuming that the rate of adsorption is proportional to the number of unoccupied adsorption sites. In this model, the difference between the amount of adsorbate adsorbed at equilibrium (*q*_e_) and at a given time (*q*_*t*_) is used to determine the adsorption rate, which depends primarily on the adsorbent's properties. The *t*–*q*_*t*_ plot for the PFO model is shown in Fig. 4b. In contrast, the PSO model assumes that chemisorption, involving electron sharing or exchange between the adsorbate and the adsorbent, is the rate-limiting step. The PSO model accounts for adsorption controlled by the number of available surface sites, with the adsorption rate proportional to the square of the number of unoccupied sites. The corresponding plot is presented in Fig. 4c.

The intraparticle diffusion model, proposed by Weber and Morris, is used to describe the diffusion of adsorbate molecules into the porous structure of the adsorbent, which governs the adsorption rate. The intraparticle diffusion plot is shown in Fig. 4d.

As summarized in Table 1, comparison of the correlation coefficients (*R*^2^) indicates that the PFO model does not adequately describe the adsorption kinetics, whereas the PSO model provides a superior fit. This suggests that the adsorption of Pb^2+^ onto Fe_3_O_4_/AC is predominantly controlled by chemisorption, involving covalent interactions and ion exchange between the pollutant and the adsorbent. Therefore, the PSO model is used in this study to describe the chemical adsorption process.^38,39^

**Table 1:** Fitting results of linear isotherms for the adsorption of Pb(ii) by Fe_3_O_4_/AC nanocomposite

Model	Parameter	*R* ^2^
Pseudo-first-order	*q*_e_ (mg g^−1^) = 206.43 ± 1.005	0.3325
	*K*_1_ (L mg^−1^) = −0.0000068 ± 0.000003	
	*q*_*t*_ (mg g^−1^) = 123.45 ± 0.00005	
Pseudo-second-order	*K*_2_ (L mg^−1^) = 0.03 ± 0.002	0.99994
	*q*_e_ (mg g^−1^) = 123.48 ± 0.00005	
Intraparticle diffusion	*K*_d_ = 0.70 ± 0.09	0.9293
	*C* = 117.40 ± 0.5	
Langmuir	*b* (L mg^−1^) = 0.857 ± 0.0001	0.97061
	*q*_m_ (mg g^−1^) = 833.33 ± 0.0004	
Freundlich	*n* = 2.46 ± 0.02	0.99453
	*K*_F_ (L mg^−1^) = 329.32 ± 1.028	
Temkin	*B* (J mol^−1^) = 129.8 ± 17.4	0.9653
	*K*_T_ (L mg^−1^) = 18.11 ± 1.8	

### 3.3. Effect of adsorbent dose

Fig. 4e shows the effect of adsorbent dosage on Pb^2+^ removal efficiency and capacity, with experiments conducted at pH 9, an initial Pb^2+^ concentration of 50 mg L^−1^, and a contact time of 30 minutes. Increasing the adsorbent dose from 1 to 6 mg led to an increase in removal efficiency and a decrease in adsorption capacity. The highest removal efficiency of >99.5% was observed at a dose of 3 mg, corresponding to an adsorption capacity of 248.97 mg g^−1^. While increasing the adsorbent dose provides more available adsorption sites, the total amount of Pb^2+^ ions in the solution remains constant. Consequently, the observed decrease in adsorption capacity with higher adsorbent doses can be attributed to the distribution of a fixed amount of lead over a larger number of active sites.

### 3.4. Effect of initial concentration

The effect of initial Pb^2+^ concentration on adsorption was investigated over the range of 50–210 mg L^−1^ under the previously determined optimal conditions. As the initial concentration increases, the fixed number of active sites on the adsorbent surface becomes progressively occupied by Pb^2+^ ions and the formed precipitate. As shown in Fig. 4f, the removal efficiency decreases with increasing initial concentration, while the adsorption capacity increases. The highest removal efficiency of >99.5% and an adsorption capacity of 249.24 mg g^−1^ were observed at an initial concentration of 50 mg L^−1^. These results indicate that as Pb^2+^ concentration increases, the available surface area and adsorption sites become saturated, leading to a reduction in removal efficiency.^40,41^ To further validate these findings, complementary characterizations including XRD, VSM, and XPS were conducted.

The XRD patterns of the Fe_3_O_4_/AC nanocomposite before and after Pb^2+^ removal at two initial concentrations (50 and 210 mg L^−1^) are shown in Fig. 4g. The XRD results confirm the formation of 2PbCO_3_·Pb(OH)_2_ at both concentrations.^42^ As evident from the figure, the intensity of the peaks corresponding to this precipitate phase increases with increasing Pb^2+^ concentration, indicating greater deposition of lead compounds on the adsorbent surface. The adsorption capacity also increases with higher initial concentrations, as more Pb^2+^ ions occupy the available active sites on the nanocomposite. On the other hand, the *M*_S_ of Fe_3_O_4_/AC after Pb^2+^ adsorption at initial concentrations of 50 and 210 mg L^−1^ was measured to be 10.56 and 7.96 emu g^−1^, respectively, as shown in Fig. 4h. The decrease in *M*_S_ is attributed to the formation of a non-magnetic lead-containing phase on the nanocomposite surface. Despite this reduction, the Fe_3_O_4_/AC nanocomposite retains sufficient magnetization for rapid separation from aqueous solutions using external magnets.

The XPS spectra of the Fe_3_O_4_/AC nanocomposite before and after Pb^2+^ adsorption are presented in Fig. 5a. The characteristic peaks corresponding to oxygen, carbon, and iron are clearly observed in both spectra. Additional peaks after adsorption are attributed to lead, specifically the Pb4f orbital. The high-resolution Fe 2p spectra of Fe_3_O_4_/AC before and after Pb^2+^ removal at *C*_0_ = 50 and 210 ppm are shown in Fig. 5b. Two main spin–orbit components corresponding to Fe 2p_3/2_ (≈708–712 eV) and Fe 2p_1/2_ (≈722–725 eV) are observed, with a spin–orbit splitting of approximately 13 eV, confirming the preservation of the spinel ferrite structure after batch experiments. Deconvolution of the Fe 2p_3/2_ peak demonstrates the coexistence of Fe^2+^ and Fe^3+^ species, reflecting the intrinsic mixed-valence nature of the ferrite phase. The component centered at ∼709–710 eV is attributed to Fe^2+^, while the peak located at ∼710.5–711.5 eV corresponds to Fe^3+^. A characteristic satellite feature around ∼718 eV further supports the presence of Fe^3+^ species within the lattice.^43^ For the pristine nanocomposite (before adsorption), the relative intensities of Fe^2+^ and Fe^3+^ indicate a stable ferrite structure with accessible surface-active sites. After exposure to Pb^2+^ solution at an initial concentration of 50 ppm, a relative decrease in the Fe^2+^ contribution and a slight increase in the Fe^3+^ fraction were observed, accompanied by mild attenuation of the overall Fe signal intensity. These changes suggest that Fe^2+^ sites participate in the adsorption process, likely through surface complexation between Pb^2+^ ions and Fe–OH functional groups. The interaction may induce local electron redistribution around iron centers, leading to a partial shift in the Fe^2+^/Fe^3+^ ratio. The slight reduction in peak intensity can be attributed to partial coverage of iron surface atoms by adsorbed Pb species. At a higher Pb^2+^ concentration of 210 ppm, the spectral changes become more pronounced. A further decrease in the Fe^2+^ component, an increase in the relative Fe^3+^ fraction, and significant attenuation of the Fe signal intensity are evident. This behavior indicates a concentration-dependent involvement of iron surface sites in the removal process. The enhanced modification of the Fe^2+^/Fe^3+^ ratio suggests stronger surface interactions and more extensive electronic perturbation at higher Pb loading. In addition to surface complexation, the marked decrease in Fe signal intensity at 210 ppm implies that partial surface precipitation of hydroxylated Pb species may occur at elevated concentrations. Such precipitation likely takes place on Fe–OH active sites, leading to surface coverage of iron atoms and attenuation of the photoelectron signal. Rather than a direct redox transformation of Pb^2+^, the observed spectral variations are more reasonably attributed to inner-sphere complex formation combined with possible surface precipitation of Pb–OH species under high local supersaturation conditions.

**Fig. 5:**
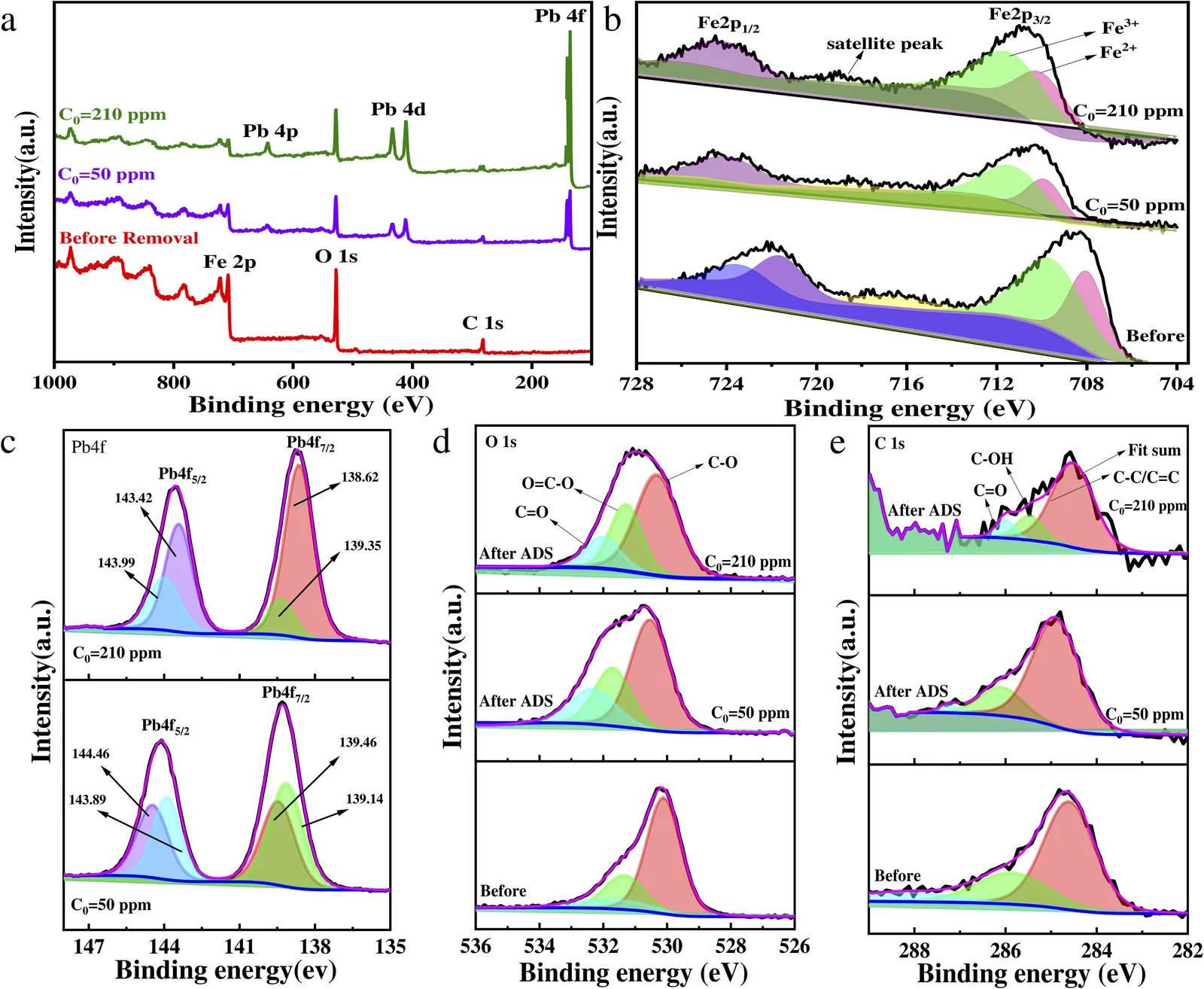
(a) Survey XPS spectra of Fe_3_O_4_/AC before and after Pb^2+^ removal. High-resolution spectra for (b) Fe 2p, (c) Pb 4f, (d) O 1s, and (e) C 1s before and after Pb^2+^ removal at concentrations of 50 and 210 ppm.

Fig. 5c presents the high-resolution Pb 4f XPS spectra of the Fe_3_O_4_/AC nanocomposite after Pb(ii) adsorption at initial concentrations of 50 and 210 ppm. The spectra exhibit the characteristic Pb 4f_7/2_ and Pb 4f_5/2_ spin–orbit doublets. Compared with 50 ppm, the results at 210 ppm show a higher Pb signal intensity together with slight peak broadening, indicating increased Pb loading and a more heterogeneous surface chemical environment.

As summarized in Table 2, each Pb 4f spectrum was successfully deconvoluted into four fitted components. The peaks located at 138.62–139.46 eV (Pb 4f_7/2_) and 143.42–144.46 eV (Pb 4f_5/2_) were assigned to Pb(ii)–O surface species and Pb(ii)–OH/PbCO_3_·Pb(OH)_2_ surface species, respectively. The corresponding binding energy (BE), full width at half maximum (FWHM), relative peak area (%), and chemical assignments are listed in Table 2.

**Table 2:** Pb 4f peak (XPS) deconvolution parameters for Fe_3_O_4_/AC after Pb^2+^ removal at *C*_0_ = 50 and 210 ppm

Sample	Component	BE (eV)	FWHM (eV)	Area (%)	Assignment
*C*_0_ = 50 ppm	Pb 4f_5/2_	144.46	1.47	21.15	Pb(ii)–O surface species
	Pb 4f_7/2_	139.46	1.55	25.74	Pb(ii)–O surface species
	Pb 4f_5/2_	143.89	1.33	21.73	Pb(ii)–OH/PbCO_3_·Pb(OH)_2_ surface species
	Pb 4f_7/2_	139.14	1.54	31.38	Pb(ii)–OH/PbCO_3_·Pb(OH)_2_ surface species
*C*_0_ = 210 ppm	Pb 4f_5/2_	143.42	1.28	28.05	Pb(ii)–O surface species
	Pb 4f_7/2_	138.62	1.35	47.50	Pb(ii)–O surface species
	Pb 4f_5/2_	143.99	1.46	15.65	Pb(ii)–OH/PbCO_3_·Pb(OH)_2_ surface species
	Pb 4f_7/2_	139.35	1.20	8.80	Pb(ii)–OH/PbCO_3_·Pb(OH)_2_ surface species

Compared with the 50 ppm sample, the Pb 4f components of the 210 ppm sample exhibit a slight shift toward lower binding energies, suggesting a change in the local chemical environment of Pb due to stronger interactions with oxygen-containing surface functional groups. A slight decrease in the FWHM values for some components indicates a more homogeneous chemical environment for the predominant Pb species. Moreover, the relative peak area of the Pb(ii)–O species increases, whereas that of the Pb(ii)–OH/PbCO_3_·Pb(OH)_2_ species decreases, indicating that surface complexation through Pb–O bonding becomes the dominant immobilization mechanism at higher Pb(ii) concentrations, while hydroxyl carbonate species remain as secondary surface products.

The identification of PbCO_3_·Pb(OH)_2_ species is consistent with the XRD results, which confirm the formation of lead hydroxycarbonate precipitates after Pb(ii) adsorption.^42,44^

The O 1s spectra of Fe_3_O_4_/AC before and after Pb^2+^ removal at concentrations of 50 and 210 mg L^−1^ are shown in Fig. 5d. Before removal, three peaks were observed at 530.11, 531.34, and 531.63 eV. After removal, the corresponding peaks shifted to 530.53, 531.71, and 532.32 eV for 50 mg L^−1^, and 530.32, 531.29, and 532.01 eV for 210 mg L^−1^, which can be assigned to C–O, O=C–O, and C=O functional groups.^45^

The C 1s spectra (Fig. 5e) before and after Pb^2+^ removal show three main components corresponding to C–C, C–OH, and C=O bonds. Before removal, the peaks were observed at 284.61, 285.86, and 288.27 eV. After experiments at 50 and 210 mg L^−1^, the peaks shifted to 284.89, 286.10, 287.23 eV, and 284.51, 285.47, 286.01 eV, respectively. The proportion of C–OH decreased from 25.89% to 18.98% and 14.16% after lead removal, while the C=O content decreased from 10.94% to 7.28% and 2.27% for the two concentrations. These changes indicate that hydroxyl and carboxyl groups act as the primary active sites for Pb^2+^ removal.^46^

Moreover, surface morphology changes observed after batch experiments, including increased compaction and decreased porosity, confirm the successful deposition of lead ions on the nanocomposite surface.^47^ These structural and chemical modifications provide strong evidence for the interaction between Pb^2+^ and the functional groups of Fe_3_O_4_/AC, including formation of white lead.

Fig. 6 presents the SEM micrographs, EDS data, and corresponding elemental mapping of the Fe_3_O_4_/AC nanocomposite after Pb^2+^ removal. The results clearly demonstrate the successful deposition of lead on the composite surface, confirming the high Pb^2+^ removal capacity of the synthesized magnetic nanocomposite from aqueous solutions. The elemental mapping and EDS results show the presence of C, Fe, O, and Pb in both samples. Based on the EDS results, at a higher concentration (210 ppm), the intensities of the C, Fe, and O peaks decrease, and the Pb peak intensity increases, which is consistent with the XRD and XPS results.

**Fig. 6:**
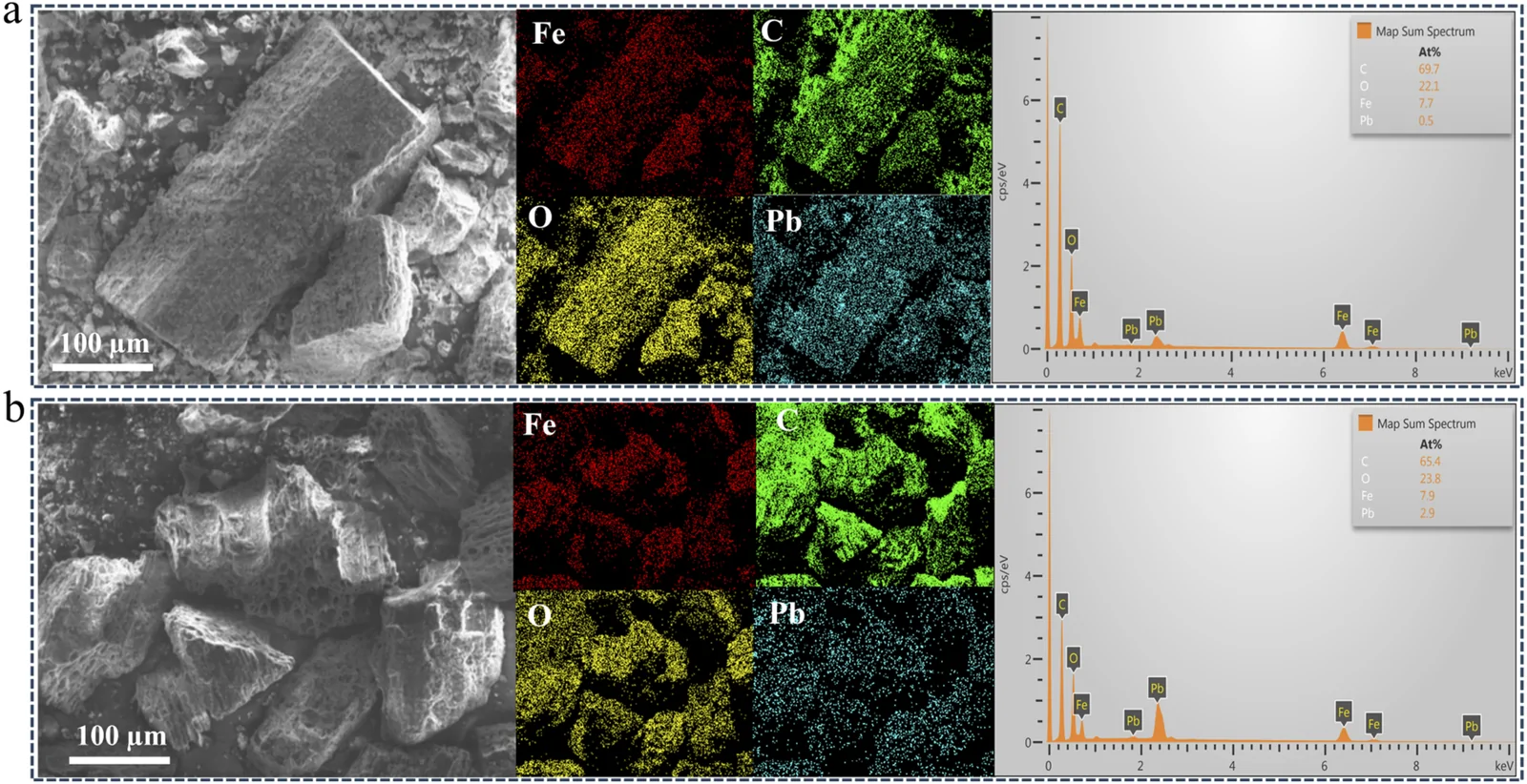
SEM images of Fe_3_O_4_/AC nanocomposite and the corresponding elemental mapping after Pb(ii) removal from contaminated water with initial lead concentrations of (a) 50 and (b) 210 ppm.

### 3.5 Adsorption isotherms

#### 3.5.1 Linear isotherm

Adsorption isotherm models are essential for describing the interaction between the adsorbent and adsorbate and for elucidating the adsorption mechanisms. In this study, the equilibrium adsorption data were analyzed using the Freundlich, Langmuir, and Temkin isotherm models, as shown in Fig. 7a–c, respectively. The Freundlich isotherm is an empirical model applied to multilayer adsorption on heterogeneous surfaces, while the Langmuir model assumes monolayer adsorption on a structurally homogeneous adsorbent with identical and energetically equivalent sites.^48^ The correlation coefficients (*R*^2^) in Table 1 indicate that the adsorption data fit the Freundlich isotherm model most closely, suggesting that Pb^2+^ adsorption occurs on multilayered, heterogeneous surfaces of the Fe_3_O_4_/AC nanocomposite.

**Fig. 7:**
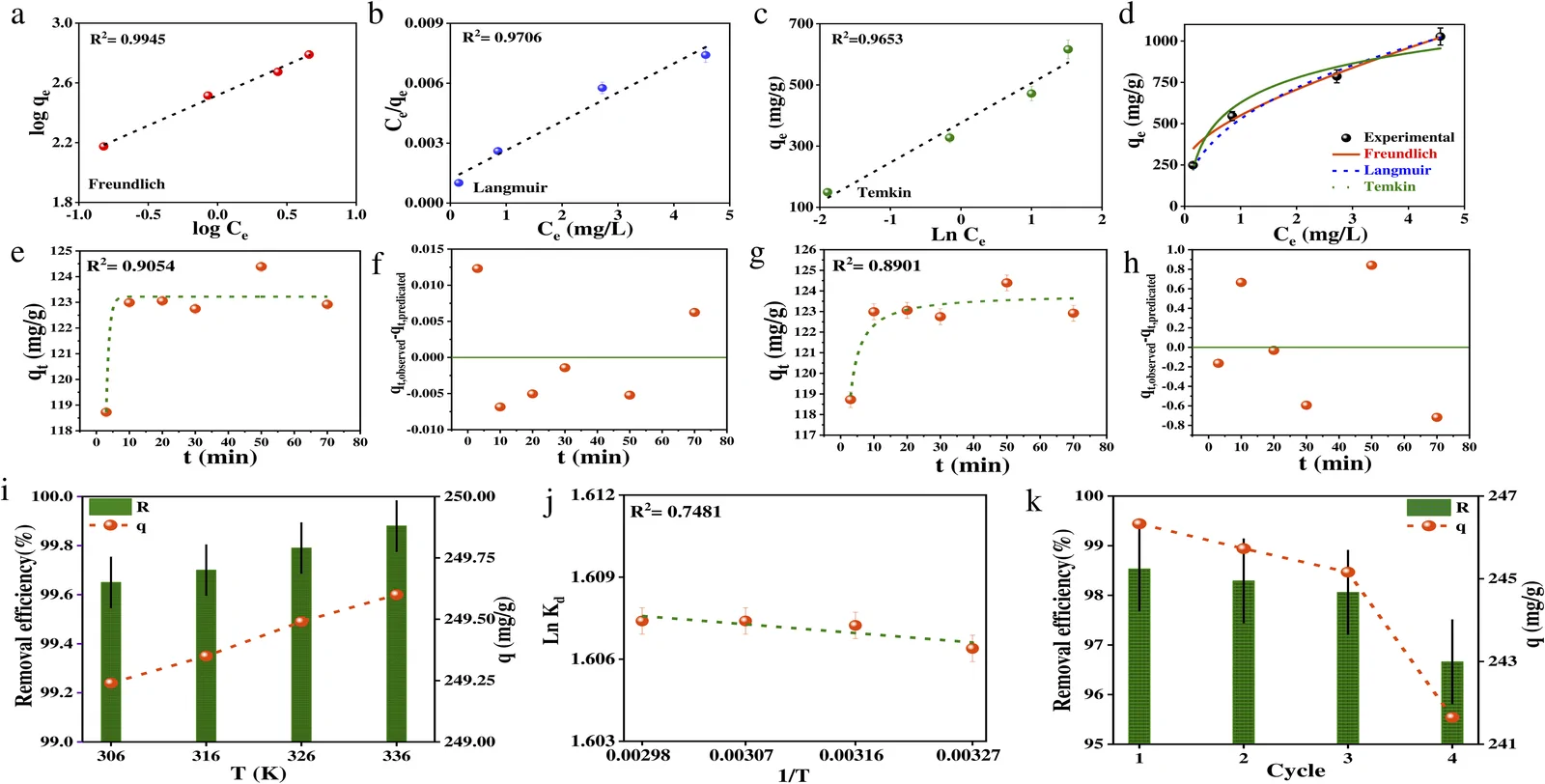
Linear fitting of the data using (a) Freundlich, (b) Langmuir, and (c) Temkin isotherms, (d) nonlinear fitting using the same isotherms, (e) nonlinear fitting of the PFO kinetic model and (f) its corresponding residual plot, (g) nonlinear fitting of the PSO kinetic model and (h) its corresponding residual plot and AIC value, (i) effect of temperature on the Pb^2+^ removal efficiency and adsorption capacity, (j) thermodynamics study, and (k) reusability results.

#### 3.5.2 Non-linear isotherms

To further evaluate the adsorption behavior, nonlinear isotherm models including Langmuir, Freundlich, and Temkin were applied to the equilibrium data, as shown in Fig. 7d. The correlation coefficients (*R*^2^) calculated from nonlinear fitting are listed in Table 3, allowing comparison of the models and interpretation of the biosorption process. As previously described, the Langmuir model assumes monolayer adsorption on a homogeneous surface with energetically equivalent sites, while the Freundlich model accounts for multilayer adsorption on heterogeneous surfaces.^49^ The Freundlich model is particularly useful for describing adsorption sites with partial occupancy of active sites. The Temkin model considers the effect of adsorption heat, which decreases with increasing surface coverage due to interactions between adsorbed molecules. Nonlinear fitting of the experimental data indicated that all three models describe the adsorption reasonably well; however, the Freundlich model exhibited the highest *R*^2^ value, confirming that Pb^2+^ adsorption on Fe_3_O_4_/AC primarily occurs on multilayered, heterogeneous surfaces.

**Table 3:** Nonlinear isotherm fitting results for Pb(ii) removal by Fe_3_O_4_/AC nanocomposite

Non-linear isotherm model	Parameters	*R* ^2^
Langmuir	*K*_L_ (L mg^−1^) = 0.85 ± 0.01	0.9646
	*q*_m_ (mg g^−1^) = 720 ± 3.25	
Freundlich	*n* = 0.39 ± 0.04	0.9927
	*K*_F_ (L mg^−1^) = 330.57 ± 24.8	
Temkin	*B* (J mol^−1^) = 18.11 ± 8.00	0.9653
	*K*_T_ (L mg^−1^) = 129.80 ± 17.40	
Pseudo-first-order	*K*_1_ (L mg^−1^) = 1.10 ± 0.053	0.9054
	*q*_e_ (mg g^−1^) = 123.22 ± 0.29	
Pseudo-second-order	*K*_2_ (L mg^−1^) = 0.065 ± 0.01	0.8901
	*q*_e_ (mg g^−1^) = 123.85 ± 0.38	

Non-linear regression was applied to analyze the kinetic data, showing that the PFO model describes the adsorption process slightly better than the PSO model, with *R*^2^ values of 0.905 and 0.890, respectively. The kinetic parameters calculated from the non-linear fits are summarized in Table 3. The corresponding non-linear fitting curves, along with their respective residual plots, are displayed in Fig. 7e–h. To avoid overfitting due to the small size of the kinetic dataset (*n* = 6), the corrected Akaike information criterion (AICc) was calculated as a complementary test. The AICc value for the PFO model was 0.637, which is lower than that of the PSO model (1.536), confirming that PFO is the statistically preferred model here. This preference is also supported by the physical relevance of the PFO parameters (*e.g.*, a positive rate constant) and the cleaner, more random distribution of its residuals around the zero line, as illustrated in the residual plots. Thus, while the simplified linear method initially pointed toward the PSO model, the non-linear analysis and statistical diagnostics indicate that the PFO model actually provides a more realistic representation of Pb^2+^ uptake on the Fe_3_O_4_/AC nanocomposite. Nevertheless, given the limited number of data points, these kinetic trends should be interpreted with reasonable caution.

### 3.6. Effect of temperature

As shown in Fig. 7i, the adsorption capacity of Pb^2+^ increases with increasing temperature. This behavior can be attributed to increased ion mobility and greater accessibility of adsorption sites at higher temperatures, which facilitate the adsorption process. However, excessively high temperatures are not economically favorable. Based on the results, an optimal adsorption temperature of 306 K (adsorption capacity of 249.24 mg g^−1^) was selected for subsequent experiments.

To further investigate the adsorption mechanism, thermodynamic parameters, including Δ*H*°, Δ*G*°, and Δ*S*°, were calculated for Pb^2+^ adsorption onto the Fe_3_O_4_/AC nanocomposite (Fig. 7j), and the results are summarized in Table 4. A positive Δ*H*° indicates that the adsorption process is endothermic, favoring higher temperatures. This behavior is evident from the slope and intercept of the Van't Hoff plot of ln *K*_L_*versus* 1/*T*. The positive Δ*S*° value suggests an increase in randomness at the solid–solution interface during adsorption, reflecting greater degrees of freedom for the adsorbed Pb^2+^ ions. The negative Δ*G*° values at all studied temperatures indicate that Pb^2+^ adsorption is spontaneous and thermodynamically favorable. The combination of positive Δ*H*° and Δ*S*° with negative Δ*G*° confirms that the adsorption process is endothermic, spontaneous, and becomes more favorable at elevated temperatures.

**Table 4:** Thermodynamic parameters for Pb^2+^ removal by Fe_3_O_4_/AC nanocomposite

Temperature (K)	*K*_L_	Δ*G*°	Δ*H*°	Δ*S*°	*R* ^2^
306	4.9850	−4.086	27.5156 ± 11.28	13.4473 ± 0.03	0.7481
316	4.9890	−4.223			
326	4.9898	−4.356			
336	4.9900	−4.490			

### 3.7. Reusability of the composite for Pb removal

The desorption process, which represents the reverse of metal ion adsorption, is critical for evaluating the reusability of an adsorbent. In this study, the reusability of the Fe_3_O_4_/AC nanocomposite was investigated using an initial Pb^2+^ concentration of 50 mg L^−1^, an adsorbent dose of 0.024 g, pH 9, and a contact time of 30 minutes at room temperature. Although the optimum adsorbent dosage determined from the batch adsorption experiments was 3 mg, 0.024 g of adsorbent together with a proportionally increased volume of Pb^2+^ solution was used in the reusability tests. This was because a small amount of the adsorbent is inevitably lost during repeated magnetic separation, washing, desorption, drying, and transfer processes. Therefore, both the adsorbent mass and the solution volume were proportionally increased by a factor of eight while maintaining the same adsorbent-to-solution ratio and all other experimental conditions. After each removal cycle, the adsorbent was treated with 0.1 M HCl to desorb the adsorbed Pb^2+^ ions and formed precipitate, therefore regenerating the active adsorption sites and available surface. The adsorbent was then thoroughly washed with deionized water until the residual acid was completely removed and a neutral pH was achieved, followed by drying before reuse in the next adsorption cycle. Fig. 7k presents the removal efficiency over successive adsorption–desorption cycles. The first cycle exhibited the highest removal efficiency of 98.53%. The nanocomposite retained substantial adsorption capacity over three cycles, with a slight decrease to 96.66% in the fourth cycle, indicating good reusability with only minor loss in performance.

Compared to previous studies (Table 5), the Fe_3_O_4_/AC nanocomposite demonstrates high adsorption capacity with a small adsorbent dose and short contact time, while maintaining structural integrity over multiple cycles. These results highlight the high stability of raspberry plant-based activated carbon, making it a promising material for practical applications in heavy metal ion removal from aqueous solutions.

**Table 5:** Comparison of the removal capacity of Pb(ii) using various adsorbent materials

Adsorbent	*q* (mg g^−1^)	pH	*t* (min)	*m* (g)	*T* (K)	*C*_0_ (mg L^−1^)	Cyc	Ref.
Sugarcane bagasse (SB)	212.31	5	120	0.1	303	400	5	50
Rice husk (TARH)	93.45	5	90	0.1	303	300	4	51
Pomegranate peel	99	5.6	120	—	300	200	—	52
Fe_3_O_4_/AC (*Prosopis juliflora*)	144.92	5	—	0.04	—	40	—	53
FeS/peanut shell	88.06	4.7	30	0.05	308	60	4	54
CSB/Fe_3_O_4_	83.33	5	105	0.5	323	50	—	55
Fe_3_O_4_/AC (raspberry bush)	249.24	9	30	0.003	303	50	4	This work

## 4. Electromagnetic wave absorption

Following the evaluation of the Fe_3_O_4_/AC nanocomposite for water remediation, its potential as a microwave absorber was investigated. The microwave absorption performance was assessed by measuring the complex relative permittivity (*ε*_r_ = *ε*′ − *jε*″) and complex relative permeability (*µ*_r_ = *µ*′ − *jµ*″) over the frequency range 12.4–18 GHz. The real components (*ε*′ and *µ*′) represent the material's ability to store electric and magnetic energy, respectively, while the imaginary components (*ε*″ and *µ*″) correspond to the energy loss or dissipation within the material, which is typically converted into heat or other forms of energy.

As illustrated in Fig. 8a, the biomass-derived activated carbon composite exhibits distinct dielectric behavior, reflecting its strong interaction with electromagnetic waves. The real permittivity (*ε*′) remains within around 6.5–7.2 across the Ku band, confirming its considerable polarization ability and efficient storage of electrical energy under an alternating field. As can be seen, the value of *ε*′ at low frequencies is approximately 7.2 and gradually decreases to approximately 6.5 with increasing frequency. This gradual decrease indicates that with increasing frequency, the material's ability to be polarized decreases, as the dipoles in the material's structure do not have sufficient time to align with the alternating electric field at higher frequencies. However, the relatively high value of *ε*′ in the entire range indicates the high ability of the material to store electrical energy, which can be due to the porous structure of activated carbon, the presence of polar functional groups, and the appropriate electronic conductivity in the carbon network. In parallel, the imaginary permittivity (*ε*″), ranging between 0.54 and 0.70 in the Ku band, demonstrates noticeable dielectric losses arising from relaxation effects and electrical conduction. The coexistence of relatively high *ε*′ and moderate *ε*″ indicates that interfacial polarization at carbon–void boundaries, defect-induced dipoles, and electron hopping within the carbon network are actively involved in energy dissipation. Minor oscillations observed in the plot are likely associated with Maxwell–Wagner–Sillars polarization, which is common in heterogeneous porous carbon-based materials. Although a higher *ε*′ enhances polarization strength, it may also deviate the input impedance from that of free space, thereby reducing absorption efficiency at certain frequencies. This drawback can be mitigated through proper filler loading or the incorporation of magnetic components. The improved dielectric response is further attributed to the homogeneous dispersion of Fe_3_O_4_ nanoparticles on the carbon surface, which promotes interfacial polarization and generates additional active sites within the porous framework. Moreover, the porous texture allows air entrapment, and the resulting dielectric contrast at air-material interfaces, which provides extra polarization, collectively enhancing the microwave absorption capacity of the composite.

**Fig. 8:**
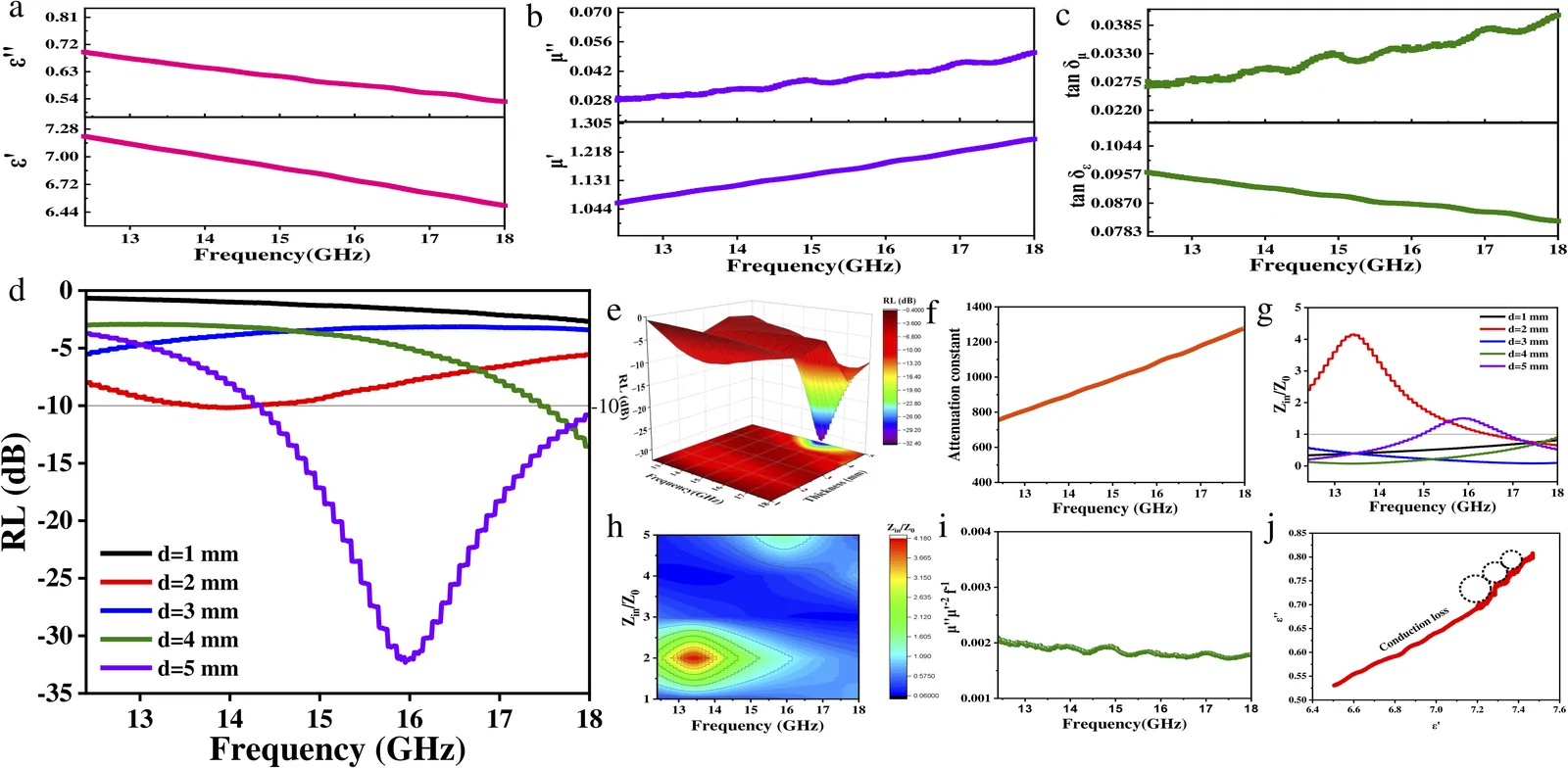
(a) Frequency-dependent behavior of the real (*ε*′) and imaginary (*ε*″) components of permittivity in Fe_3_O_4_/AC, (b) real (*µ*′) and imaginary (*µ*″) parts of permeability as a function of frequency, (c) loss tangents for dielectric (tan *δ*_ε_) and magnetic (tan *δ*_µ_) properties, (d) RL_min_ curves of the absorber at different thicknesses, (e) corresponding three-dimensional contour plots, (f) attenuation constant *α*, (g) Normalized input impedance (*Z*_in_/*Z*_0_) of the absorber, (h) 2-D mapping of *Z*_in_/*Z*_0_, and (i) *C*_0_ value in Ku band, (j) Cole–Cole plot (*ε*″ *versus ε*′) of the nanocomposite.

The magnetic permeability spectra (Fig. 8b) provide valuable additional evidence regarding the absorption mechanism. As can be seen, the real part of the magnetic permeability (*µ*′) varies within a relatively moderate range between 1.0 and 1.3 and shows a gentle and increasing trend with increasing frequency. In contrast, the imaginary part of the permeability (*µ*″) remains close to zero across the entire frequency range, indicating low magnetic loss. This behavior confirms the limited magnetic contribution of Fe_3_O_4_, consistent with the predominantly dielectric nature of the absorption. No pronounced magnetic-resonance features are observed within the measured Ku band. Moreover, very low values of *µ*″ indicate that magnetic loss processes such as spin relaxation, domain wall resonance, or eddy current loss are not very important in this system. As a result, the carbon nanocomposite framework mainly contributes to the absorption of microwaves through dielectric paths. The presence of Fe_3_O_4_ magnetic nanoparticles in the composite provides additional magnetic loss, creating a synergistic effect that enhances the overall absorption efficiency by combining both dielectric and magnetic mechanisms.^56^

As can be seen in Fig. 8c, the dielectric loss tangent (tan *δ*_ε_) in the frequency range of 12.4 to 18 GHz shows a uniformly decreasing trend, so that its value decreases from approximately 0.096 at 12.4 GHz to approximately 0.078 at 18 GHz. These changes indicate that with increasing frequency, the ability of the material to store and dissipate electrical energy gradually decreases, because at higher frequencies, the polarization of dipoles and the response of free charges to an alternating electromagnetic field weaken. However, the relatively high values of tan *δ*_ε_ in the entire frequency range indicate the presence of a significant dielectric loss in the nanocomposite structure, which is directly related to its structural properties and chemical composition. In this structure, the presence of a conductive carbon skeleton causes charge carriers to oscillate under the applied field and dissipate part of the electromagnetic energy as heat through ohmic loss. The porous carbon framework provides abundant heterogeneous interfaces that promote interfacial polarization, thereby increasing the imaginary component of permittivity (*ε*″). On the other hand, the presence of hetero atoms such as oxygen and nitrogen in the activated carbon network, along with structural defects and surface dipoles, provides the basis for the relaxation of local dipoles, which plays an important role in increasing the dielectric loss. Therefore, the gradual decrease of tan *δ*_ε_ while maintaining relatively high values indicates the electromagnetic stability of the material and the establishment of a balance between the storage and dissipation of electrical energy. In contrast, the value of the magnetic loss tangent (tan *δ*_µ_) is very low across the entire frequency range, indicating that magnetic losses are limited in this nanocomposite. Although Fe_3_O_4_ nanoparticles are present, their relatively small fraction and the frequency range considered result in a negligible magnetic contribution. Therefore, the microwave absorption is predominantly governed by dielectric loss mechanisms, including interfacial polarization, electronic conduction, and dipole relaxation.^57^

Overall, the observed behavior of tan *δ*_ε_ as a function of frequency indicates that the synthesized nanocomposite has efficient dielectric loss throughout the Ku band. These features are particularly important in the design of lightweight and carbon-based microwave absorbers, because the porous carbon structure, while providing electron conduction paths and multiple polarized regions, allows for effective absorption of waves and their conversion to heat. Precise control of porosity, functional groups, and graphitization can further enhance the absorption performance. Next, to quantitatively investigate the efficiency of microwave absorption, the RL_min_ of the nanocomposite was calculated and analyzed using transmission line theory to establish the relationship between electromagnetic properties, impedance matching, and the intensity of attenuation of incoming waves. As per the transmission line theory, RL_min_ can be computed as follows:^58^21
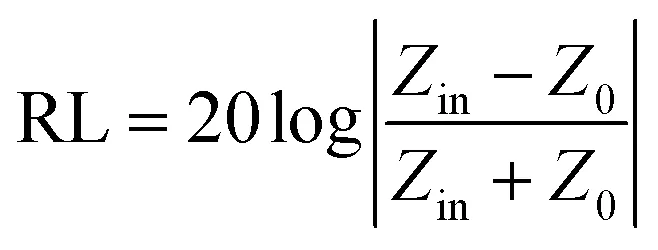
22
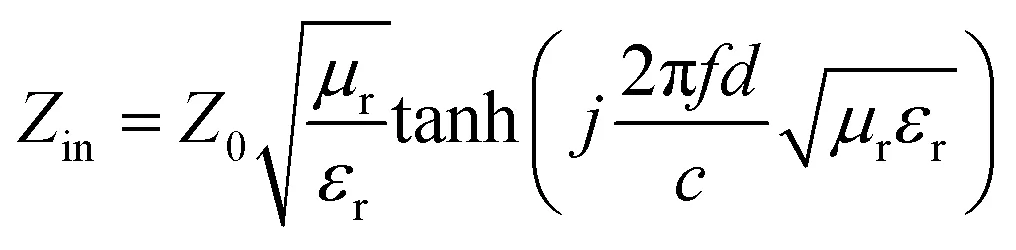
In this formulation, the RL_min_ is used to describe the extent of wave energy that is reflected, while *Z*_0_ represents the intrinsic impedance of free space (377 Ω) and *Z*_in_ corresponds to the input impedance of the absorber. The frequency of the incident electromagnetic wave is denoted by *f*, the thickness of the absorbing medium is *d*, and *c* refers to the speed of light in vacuum. When the RL value is lower than −10 dB, the absorber is generally regarded as efficient in dissipating electromagnetic energy. The frequency range over which RL < −10 dB is defined as the effective absorption bandwidth, highlighting the operational range where strong wave attenuation can be achieved.

The RL_min_ curve of the present composite demonstrates a frequency- and thickness-dependent behavior. As presented in Fig. 8d and e, the absorber exhibits strong attenuation, with RL_min_ approaching −30 dB at a thickness of 5 mm, corresponding to an absorption efficiency of approximately 99.9%. The positions of these minima shift with thickness, a trend that can be explained by the quarter-wavelength matching condition, where destructive interference between incident and reflected waves within the material enhances absorption at particular frequencies. The relatively broad absorption bands indicate that the material is capable of attenuating electromagnetic waves over several gigahertz, although the bandwidth is still limited by the absence of strong magnetic losses.^59^ It is noteworthy that even without magnetic contributions, optimized thickness combined with dielectric dissipation yields significant attenuation, which highlights the promise of biomass-derived carbon as eco-friendly and cost-effective microwave absorbers. Nevertheless, further improvements in both the depth of RL_min_ and bandwidth require better impedance matching through a balance of dielectric and magnetic parameters.^60^ According to transmission line theory, the attenuation constant *α* can be expressed as:23
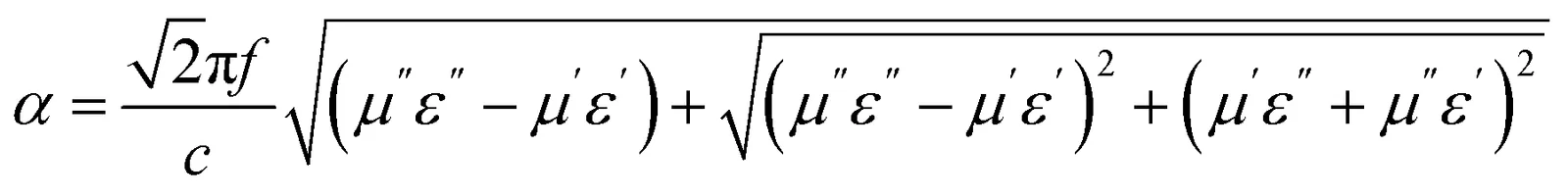


Fig. 8f illustrates the attenuation constant of the activated-carbon nanocomposite across the 12.4 to 18 GHz frequency range. As is evident, *α* exhibits a clear and nearly linear monotonic increase with increasing frequency. The *α* value commences at approximately 750 at 12.4 GHz and significantly increases to over 1250 at 18 GHz. This behavior signifies that the intrinsic capacity of the material to dissipate electromagnetic energy by converting it to heat, and consequently, the effective attenuation of the incident waves, is substantially enhanced at higher frequencies. The increase in the attenuation constant is directly attributed to dielectric loss mechanisms, with a minor contribution from the small magnetic losses of Fe_3_O_4_ nanoparticles. This trend underscores that as the frequency increases, the contributions of the dissipation processes, particularly various polarization relaxations (such as interfacial and dipolar polarization) and conduction losses (arising from electrical currents within the carbon matrix), become increasingly prominent. Consequently, the attenuation constant is an essential metric for assessing the material's internal loss capacity and its ability to absorb wave energy before it reaches the back surface of the absorber.

The normalized input impedance analysis provides direct evidence of the relationship between intrinsic material properties and external reflection. As shown in Fig. 8g and h, the absorber achieves optimal performance when the impedance ratio (*Z*_in_/*Z*_0_) approaches unity, signifying near-perfect impedance matching with free space. At these points, the RL_min_ is observed, confirming that effective absorption requires simultaneous impedance matching and internal loss. However, deviations of *Z*_in_/*Z*_0_ from unity at other frequencies are indicative of a mismatch between the absorber and air, which leads to increased reflection rather than absorption.^61^ The microwave absorption behavior of the Fe_3_O_4_/AC nanocomposite is best understood as the outcome of a division of labor between its two constituents: the porous carbon network, which governs the dielectric response, and the Fe_3_O_4_ nanoparticles, which introduce a magnetic component that regulates the overall impedance of the composite. The carbon skeleton alone would behave as a nearly purely dielectric medium (*µ*_r_ ≈ 1), with *ε*′ in the range of 6.5–7.2 arising from its conductive, defect-rich, heteroatom-doped framework. For such a medium, the normalized input impedance *Z* = |*Z*_in_/*Z*_0_| ≈ √(*µ*_r_/*ε*_r_) is inherently pulled well below unity, since *ε*_r_ is large while *µ*_r_ contributes nothing to compensate it; the result is strong reflection of incident waves at the absorber surface before any internal dissipation can occur, regardless of how favorable the intrinsic loss tangent of the carbon may be. This is the essence of the impedance-mismatch problem faced by highly dielectric carbon-based absorbers.

The incorporation of Fe_3_O_4_ nanoparticles addresses this limitation in two complementary ways. First, at the Fe_3_O_4_–carbon interface, the difference in electronic structure between the oxide nanoparticles and the surrounding conductive carbon promotes local charge accumulation and redistribution under an alternating field, which acts as an additional interfacial-polarization center and reinforces the dielectric loss already provided by the carbon matrix. Second, and more importantly for impedance matching, Fe_3_O_4_ increases the value of *µ*′ modestly above unity (from approximately 1.0 to 1.3 across the Ku band) and contributes a small but frequency-dependent *µ*″, which, although too weak to constitute a resonant magnetic-loss channel on its own, is sufficient to shift Z back toward 1, partially offsetting the mismatch imposed by the high *ε*_r_ of carbon. This is reflected in the correspondence between the frequency range where *µ*′ is largest and the frequency range where the deepest RL_min_ and the closest *Z*_in_/*Z*_0_ ≈ 1 values are observed (Fig. 8g and h). In other words, Fe_3_O_4_ functions less as an independent loss center competing with the dielectric response of carbon, and more as an impedance-tuning agent that allows a larger fraction of the dielectrically dissipated energy to actually enter the absorber rather than being reflected at its surface. This general strategy—using a magnetic phase to correct the impedance mismatch of an intrinsically dielectric-dominant framework rather than to add a separate, resonant loss mechanism is consistent with the magneto–dielectric synergy recently reported by Wang *et al.* for Ni-decorated Ta_4_C_3_T_*x*_ MXene^62^ and by Hu *et al.*^63^ for CoNi-coated Sn whiskers. Since the Fe_3_O_4_ loading and its dispersion as isolated nanoparticles here are comparatively modest relative to those systems, the resulting magnetic contribution and hence the width of the impedance-matched frequency window remains correspondingly limited, to which we attribute the relatively narrow effective absorption bandwidth of the present composite, despite its competitive RL_min_ value. Increasing the Fe_3_O_4_ loading or promoting more intimate Fe_3_O_4_–carbon heterointerfaces are therefore identified as the most direct routes for broadening the bandwidth of this biomass-derived absorber in future work.

Fig. 8i shows the frequency dependence of the magnetic loss *C*_0_, which is described as:

24 *C*_0_ = *µ*″*µ*′^−2^*f*^−1^

In an ideal case, where magnetic loss is caused solely by eddy currents, *C*_0_ would remain constant across the frequency range. In our sample, however, a slight frequency dependence of *C*_0_ is observed, indicating that magnetic losses are not exclusively controlled by eddy currents. Nevertheless, the nearly constant value of the real permeability (*µ*′) and the very low value of the imaginary permeability (*µ*″) suggest that magnetic resonance contributes only marginally to microwave absorption. The highly porous structure of the activated-carbon nanocomposite further enhances absorption. Internal pores induce multiple reflections and scattering of electromagnetic waves, effectively increasing the propagation path and promoting energy dissipation. Additionally, the interconnected carbon network facilitates the conversion of electromagnetic energy into localized currents, while interfacial interactions between the conductive carbon framework and any magnetic domains create heterogeneous charge distributions that enhance dielectric losses. Together, the porous architecture and interfacial effects improve impedance matching, resulting in highly efficient wave absorption.

To further elucidate the origin of dielectric loss, Cole–Cole plots (*ε*″ *versus ε*′) were constructed based on Debye relaxation theory. According to this theory, each semicircle in the *ε*′ − *ε*″ plot corresponds to a distinct polarization relaxation process, which can be described by the following relationship: (*ε*′ − (*ε*_s_ + *ε*_∞_)/2)^2^ + (*ε*″)^2^ = ((*ε*_s_ − *ε*_∞_)/2)^2^, where *ε*_s_ and *ε*_∞_ are the static and high-frequency permittivity, respectively. As shown in Fig. 8j, the Cole–Cole plot is dominated by an essentially linear trend across most of the *ε*′ range (6.4–7.2), which is characteristic of conduction loss arising from electron hopping and migration within the interconnected conductive carbon network. Only at the high-frequency end of the plot (*ε*′ ≈ 7.2–7.5) does the curve deviate from linearity, exhibiting three closely overlapping and distorted arcs rather than well-resolved semicircles. This overlap indicates the coexistence of multiple polarization relaxation processes within this narrow *ε*′ range, tentatively identified as follows: (1) interfacial polarization (Maxwell–Wagner–Sillars polarization), arising from charge accumulation at heterogeneous interfaces, including carbon–void boundaries, Fe_3_O_4_–carbon heterointerfaces, and defect sites within the carbon matrix; (2) dipolar polarization from polar functional groups (*e.g.*, C=O, C–O, and O–H) on the carbon surface; and (3) dipolar polarization associated with structural defects (*e.g.*, vacancies and dangling bonds) within the carbon framework. Since these three relaxation processes possess similar relaxation time constants, their corresponding semicircular arcs heavily overlap and cannot be individually resolved, appearing instead as a cluster of distorted arcs superimposed on the dominant conduction-loss background.^64–66^

Overall, the excellent attenuation performance of the activated-carbon nanocomposite arises from a synergy of dielectric dissipation and interfacial interactions, with only a minor contribution from magnetic losses. To assess the actual impact of electromagnetic absorption on scattering behavior, the monostatic RCS of the absorber-coated structure was compared with that of a perfect electric conductor (PEC) reference, as illustrated in Fig. 9a. According to the results, the absorber-coated structure exhibits a pronounced reduction in the main RCS peak relative to the PEC target, with a maximum reduction reaching approximately 38.5 dB m^2^. This substantial decrease clearly indicates that the proposed absorbing structure is not merely effective in suppressing normal-incidence reflection, but also significantly attenuates the dominant specular scattering components that primarily contribute to monostatic RCS. From an electromagnetic perspective, this behavior is closely associated with the suppression of in-phase surface currents and the consequent reduction in the amplitude of the backscattered field along the transmitter–receiver direction. Such a mechanism is widely exploited in the design of next-generation radar-absorbing coatings for stealth and low-observability applications.^67^

**Fig. 9:**
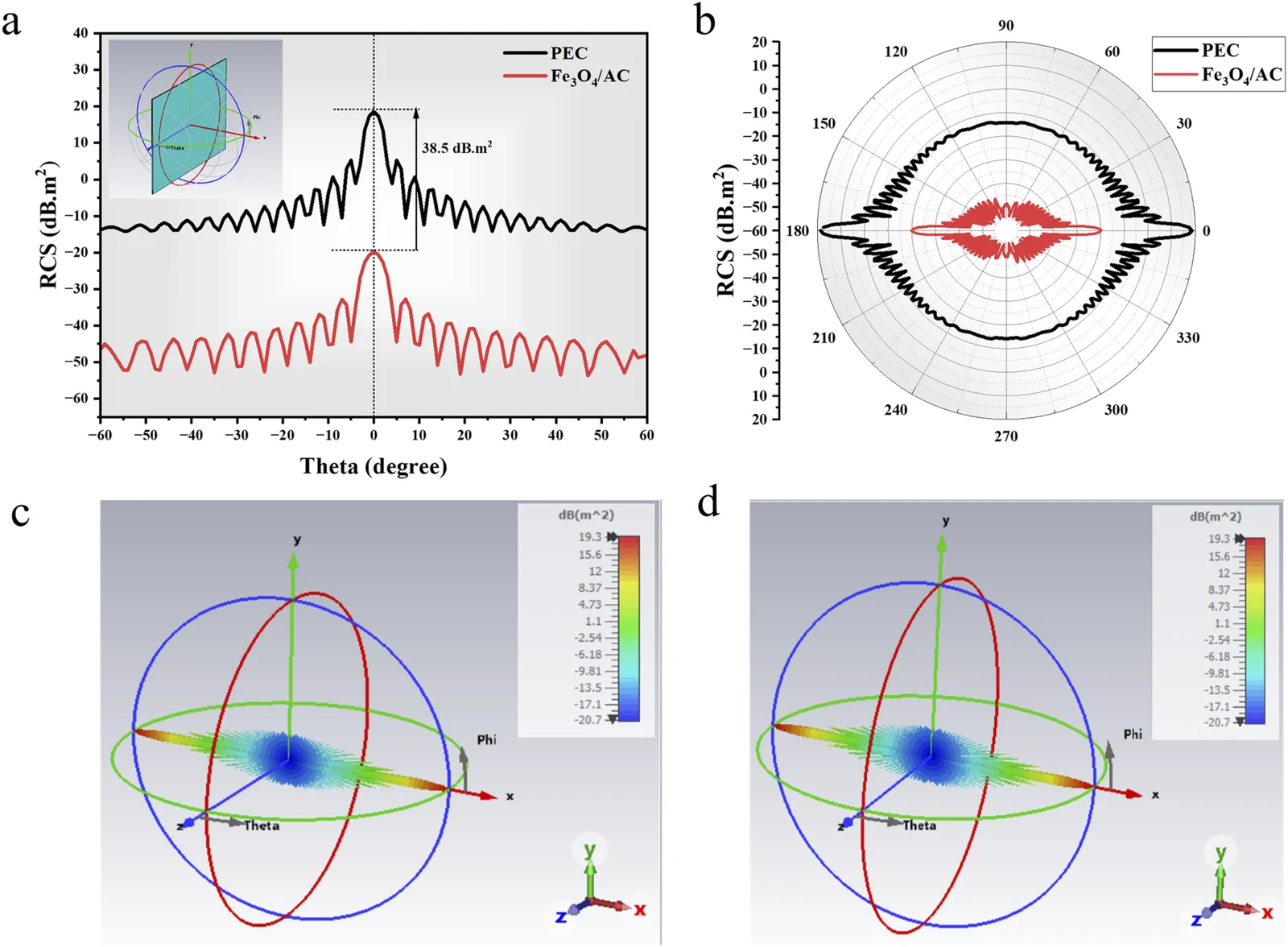
(a) Schematic of the PEC plate coated with the Fe_3_O_4_/AC absorber and comparison of the 3D RCS simulation results for the uncoated and coated PEC plates, (b) polar-coordinate RCS pattern of the PEC plate coated with Fe_3_O_4_/AC, (c) 3D RCS simulation results of the uncoated PEC plate, (d) 3D RCS simulation results of the PEC plate coated with Fe_3_O_4_/AC.

The two-dimensional polar RCS patterns presented in Fig. 9b provide detailed insight into the angular distribution of scattered energy for both the PEC reference and the absorber-coated structure. In the case of the PEC reference, a narrow and high-amplitude main lobe is observed, which is characteristic of strong specular scattering and coherent reflection in the incident direction. In contrast, the introduction of the absorbing structure results in a marked reduction in the main lobe amplitude accompanied by a noticeable broadening of the angular scattering distribution.

This modification in the scattering pattern suggests that the incident electromagnetic energy, instead of being concentrated in a specular direction, is either dissipated within the absorber volume or redistributed as diffuse scattering over a wide range of angles. Such angular redistribution is a critical strategy for reducing radar detectability, particularly under oblique incidence conditions where RL_min_ alone is insufficient to fully characterize stealth performance.^68,69^

To achieve a more comprehensive understanding of the scattering characteristics, three-dimensional RCS distributions in the *θ*–*φ* angular space are presented in Fig. 9c and d. For the PEC reference, the RCS pattern exhibits a high degree of symmetry, with pronounced maxima aligned with the incident direction, indicating the dominance of specular scattering mechanisms. In contrast, the incorporation of the absorbing layer leads to a substantial reduction in the overall RCS magnitude and the effective elimination of high-intensity scattering regions. Beyond amplitude reduction, a clear breaking of spatial scattering symmetry is observed. This phenomenon implies that the absorbing structure induces localized modifications in the phase and amplitude of surface currents, thereby disrupting dominant scattering pathways and converting coherent reflections into weaker, spatially distributed scattered fields. This behavior is in strong agreement with recent reports on multi-scale, gradient, and metastructured absorbers that simultaneously target electromagnetic absorption and scattering control.^70^

## 5. Mechanisms of Pb(ii) removal and electromagnetic wave absorption

According to the XRD patterns, new peaks were detected as Pb hydrate or carbonate. However, the intensity of XRD peaks decreased, indicating that Pb(ii) adsorption affected the layered structure of the nanocomposite. The results showed that the adsorption of Pb(ii) onto the nanocomposite was partly mediated by a precipitation reaction.^71,72^ The bonds between the metal ions and hydroxyl groups are formed through surface complexation. The abundance of functional groups and the possible removal of Pb(ii) were mainly related to the surface complexation and electrostatic interaction of the functional groups of the nanocomposite.^73,74^ The XPS results showed that lead carbonate and hydroxide were formed during the adsorption process. The experimental results of pH and pH_PZC_ potential change indicate the presence of electrostatic interactions between the positively charged adsorbates and the remaining negatively charged oxygen-containing functional groups. The adsorption phenomenon at different pH levels was accompanied by two possible reactions. One was Pb(ii) precipitation onto the nanocomposite, and the other was surface complexation. The different fractions of Pb species in different pH environments may be an influential factor. In addition, activated carbon can adsorb metal ions with nano-sized pore structures and oxygen groups on the surface. Moreover, the carbon surface was negatively charged, which could adsorb positively charged ions such as Pb(ii) by electrostatic binding. The excess surface hydroxyl groups and carbonate ions from the inner layer cause precipitation.^75^ Therefore, the mechanism (Fig. 10) of the nanocomposite for removing Pb(ii) ions can be summarized as surface complexation, precipitation, isomorphic substitution, and physical adsorption.

**Fig. 10:**
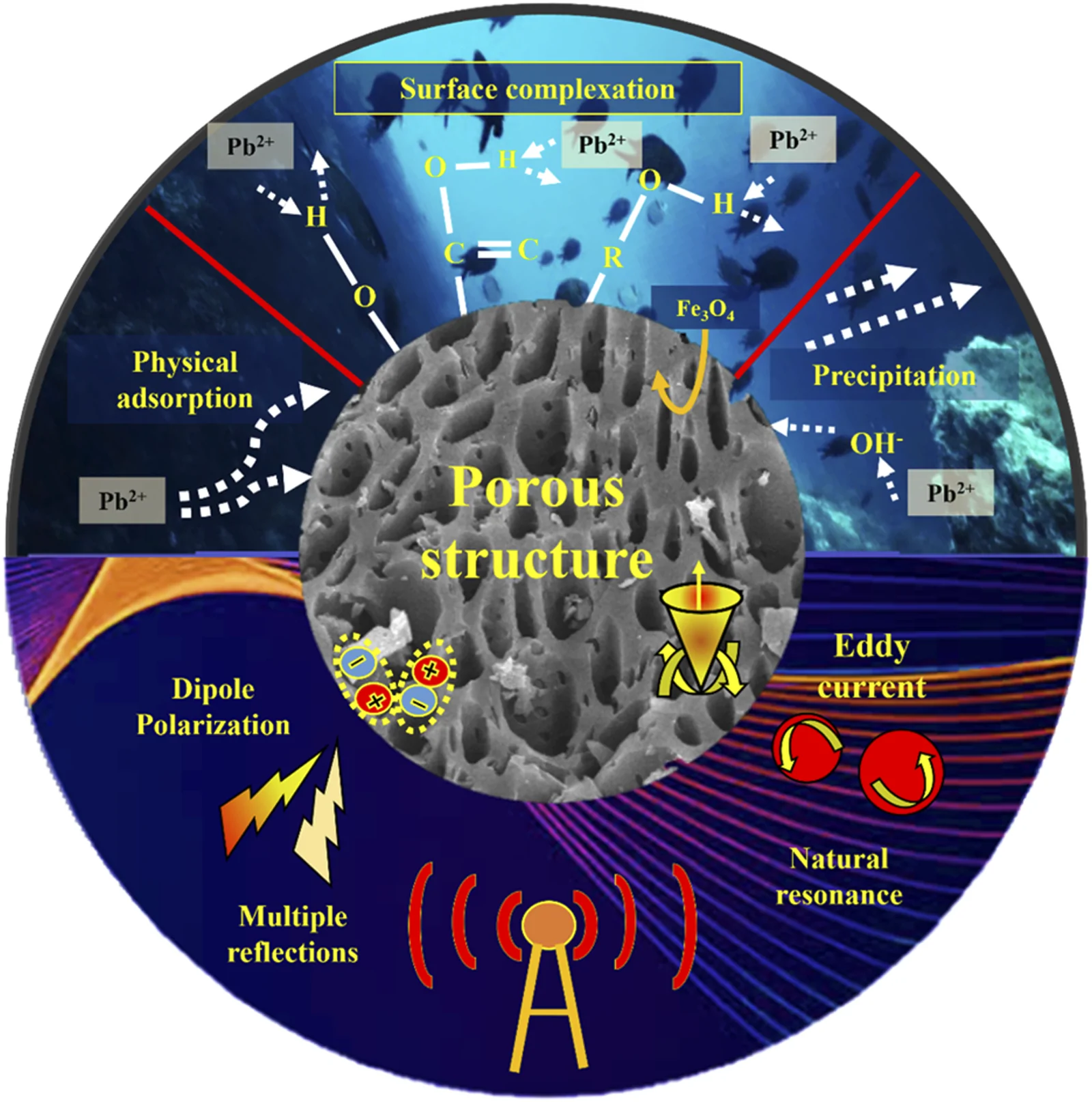
Pb^2+^ removal and EMW absorption mechanisms by Fe_3_O_4_/AC nanocomposite.

The electromagnetic wave absorption ability of the nanocomposite essentially lies in a synergistic mechanism in which dielectric losses are the main energy dissipation channel. As revealed from the complex permittivity analysis, the high energy storage capacity is due to the heterogeneous structure, which is conducive to charge accumulation at the interfaces. This process involves the field-induced rearrangement of molecular dipoles or charge carriers trapped in structural defects and interfaces in the porous carbon matrix, leading to significant energy dissipation, which is evident at the maximum value. Complementing this phenomenon is surface polarization, which enhances the dielectric response by accumulating space charges at the boundaries between the magnetic nanoparticles and the surrounding carbon phase. In contrast, the magnetic energy dissipation is minimal and non-resonant. The nearly unity true permeability value and the flat, near-zero imaginary permeability value clearly rule out the presence of significant magnetic resonance peaks (such as ferromagnetic resonance), which is consistent with the observation of the magnetic loss tangent remaining negligible over the entire measured spectrum. The observed minor magnetic contributions are attributed solely to eddy current losses due to the conductive nature of the carbon matrix, which acts as an induction loop and opposes the external magnetic field. Crucially, the high overall attenuation is achieved not only through dissipation, but also through efficient wave guidance. The porous and disordered structure acts as an efficient scattering medium and induces multiple internal reflections, which significantly increase the effective path length of the waves in the absorber layer. This enhanced scattering, combined with precise impedance matching conditions, ensures maximum energy penetration into the material, where dielectric dissipation mechanisms can effectively convert electromagnetic energy to thermal energy, leading to deep minima in reflection loss.

## 6. Conclusion

A Fe_3_O_4_/AC nanocomposite was synthesized from raspberry bush-derived activated carbon and demonstrated high efficiency in removing Pb^2+^ from aqueous solutions, achieving optimal removal at pH 9, 30 minutes, 3 mg adsorbent dose, 50 mg L^−1^ initial concentration, and 306 K. The material retained significant removal capacity over four cycles, indicating good stability and reusability. In addition, the nanocomposite exhibited effective microwave absorption, dominated by dielectric loss mechanisms, with RL_min_ values of approximately −30 dB. High permittivity, interfacial polarization, and favorable impedance matching contributed to strong attenuation, while the weak magnetic response limited the bandwidth. These results highlight the dual functionality of Fe_3_O_4_/AC nanocomposite with potential applications in water purification and electromagnetic wave attenuation.

## Author contributions

Bagher Aslibeiki: writing – review & editing, conceptualization, data curation, investigation, funding acquisition, project administration, resources, supervision, Mahsa Imani: writing – original draft, software, formal analysis, data curation, methodology, validation, Mahsa Mahmoodi: writing – review & editing, formal analysis, methodology, validation, Sagnik Ghosh: writing – review & editing, investigation, Davide Peddis: writing – review & editing, visualization, Tapati Sarkar: writing – review & editing, funding acquisition, resources, supervision.

## Conflicts of interest

There are no conflicts to declare.

## Data Availability

The authors confirm that the data supporting the findings of this study are available within the article. Raw research data are available upon request.
